# A Systems Biology Approach Identifies a Regulatory Network in Parotid Acinar Cell Terminal Differentiation

**DOI:** 10.1371/journal.pone.0125153

**Published:** 2015-04-30

**Authors:** Melissa A. Metzler, Srirangapatnam G. Venkatesh, Jaganathan Lakshmanan, Anne L. Carenbauer, Sara M. Perez, Sarah A. Andres, Savitri Appana, Guy N. Brock, James L. Wittliff, Douglas S. Darling

**Affiliations:** 1 Department of Oral Immunology and Infectious Diseases, University of Louisville, Louisville, Kentucky, United States of America; 2 Department of Biochemistry & Molecular Biology, University of Louisville, Louisville, Kentucky, United States of America; 3 Institute for Molecular Diversity and Drug Design, University of Louisville, Louisville, Kentucky, United States of America and; 4 Department of Bioinformatics and Biostatistics, University of Louisville, Louisville, Kentucky, United States of America; Centro Nacional de Investigaciones Oncológicas (CNIO), SPAIN

## Abstract

**Objective:**

The transcription factor networks that drive parotid salivary gland progenitor cells to terminally differentiate, remain largely unknown and are vital to understanding the regeneration process.

**Methodology:**

A systems biology approach was taken to measure mRNA and microRNA expression in vivo across acinar cell terminal differentiation in the rat parotid salivary gland. Laser capture microdissection (LCM) was used to specifically isolate acinar cell RNA at times spanning the month-long period of parotid differentiation.

**Results:**

Clustering of microarray measurements suggests that expression occurs in four stages. mRNA expression patterns suggest a novel role for *Pparg* which is transiently increased during mid postnatal differentiation in concert with several target gene mRNAs. 79 microRNAs are significantly differentially expressed across time. Profiles of statistically significant changes of mRNA expression, combined with reciprocal correlations of microRNAs and their target mRNAs, suggest a putative network involving *Klf4*, a differentiation inhibiting transcription factor, which decreases as several targeting microRNAs increase late in differentiation. The network suggests a molecular switch (involving *Prdm1*, *Sox11*, *Pax5*, miR-200a, and miR-30a) progressively decreases repression of *Xbp1* gene transcription, in concert with decreased translational repression by miR-214. *The transcription factor Xbp1* mRNA is initially low, increases progressively, and may be maintained by a positive feedback loop with *Atf6*. Transfection studies show that *Xbp1Mist1* promoter. In addition, *Xbp1* and *Mist1* each activate the parotid secretory protein (*Psp*) gene, which encodes an abundant salivary protein, and is a marker of terminal differentiation.

**Conclusion:**

This study identifies novel expression patterns of *Pparg*, *Klf4*, and *Sox11* during parotid acinar cell differentiation, as well as numerous differentially expressed microRNAs. Network analysis identifies a novel stemness arm, a genetic switch involving transcription factors and microRNAs, and transition to an *Xbp1* driven differentiation network. This proposed network suggests key regulatory interactions in parotid gland terminal differentiation.

## Introduction

Salivary gland dysfunction affects millions across the nation, and results in complications that can lead to a decline in oral health as well as overall quality of life [1]. Whole saliva provides many functions in the oral cavity such as defense against pathogens, lubrication for speech and digestion, and regulation of pH [2]. Without functioning salivary glands, patients suffer chronic xerostomia (dry mouth). Along with discomfort and difficulty swallowing food, these patients are at a high risk for chronic oral infections and dental caries [3]. Chronic xerostomia is a common complication for patients undergoing radiation therapy for head and neck cancer as salivary glands are especially sensitive to radiation damage [4]. Most treatment is palliative, as current treatment options that address the underlying gland dysfunction are limited [5]. The ability to regenerate or restore function to damaged glands would greatly increase patient health and quality of life [6, 7].

Current work has made headway towards this goal by focusing on gland progenitor cells. Insights from explant cultures have shown that parasympathetic nerves, which develop within the gland, are vital for maintaining epithelial progenitor cell populations during development [8]. Within the cells, up-regulation of the KIT pathway by FGFR2b signaling expands the KIT+ progenitor cell population in the end buds and also regulates progenitor cells in the ducts, through interactions with the nerves [9]. Transplantation of c-Kit+ stem cells (derived either from bone marrow or the gland itself) into the glands of irradiated mice forms acini and improves tissue function [10]. However, while much work has focused on identifying genes involved in early development during morphogenesis of the salivary glands [11, 12], regulation of the later stage of terminal differentiation remains relatively unstudied.

Differentiation of rat parotid salivary gland acinar cells occurs during the last week of gestation and the first postnatal month [13]. Just before birth, parotid acinar cells are still poorly formed. Terminal clusters do not appear to have a lumen, and no electron dense granules are present in the cytoplasm. No secretions from these clusters have been seen at these early stages. Nuclei are centrally located and the endoplasmic reticulum (ER) and Golgi are small [13]. Acinar cells mature postnatally, gaining dense granules and increasing expression of salivary cargo proteins such as amylase, parotid secretory protein (Psp/ BPIFA2), and DNase I, and becoming polarized, until at around postnatal day 25 (P25) they are considered fully mature [14].

While regulatory pathways that drive terminal differentiation are unknown, studies in knockout mice have identified two relevant transcription factors. Deficiencies in either X-box binding protein 1 (*Xbp1*) or basic helix-loop-helix family, member a15 (Bhlha15, Mist1) impairs both salivary gland and exocrine pancreas development, and while each gland still develops in knockout mice, the acinar cells are either disorganized or poorly formed. Mist1 is a basic helix-loop-helix transcription factor that is specific to serous exocrine tissue [15–17]. *Mist1* knockout mice display disorganized acinar cells at two months of age that have lost their apical/basal polarity. Secretory granules in these mice are present but without clear localization, and nuclei are no longer basally located. Hence, in the *Mist1* knockout mice the parotid gland develops, but the late stages of cellular differentiation are disrupted. *Xbp1* is an essential component of the ER stress response [18, 19], as well as directing differentiation of immunoglobulin-secreting plasma cells, dendritic cells, osteoclasts and chondrocytes [20–22]. It is involved in the biogenesis and expansion of the ER to accommodate a higher protein load [23–25], and is highly expressed in exocrine tissue including the developing salivary glands [26]. The acini of submandibular salivary glands of *Xbp1* ^-/-^; Liv^*Xbp1*^ mice are smaller than their wild type counterparts and have lower expression of amylase [24]. However, the acinar cells still have abundant secretory granules and the impact of the *Xbp1* knockout on submandibular development is much less profound than on the pancreas [20]. *Mist1* and *Xbp1* are apparently involved in acinar cell terminal differentiation. However, the regulatory network which drives differentiation, and how markers of terminal differentiation such as salivary proteins are activated, remain unknown.

This project takes a systems biology approach to understanding parotid acinar differentiation by measuring both mRNA and microRNA expression changes across terminal differentiation. Using laser capture microdissection (LCM), acinar cells were isolated from developing glands at different times during differentiation. By measuring a large portion of the transcriptome over time, global patterns were identified as well as individual transcription factors apparently of importance to the process of differentiation.

## Materials and Methods

### Rat parotid laser capture microdissection

Parotid tissue was obtained from Sprague Dawley rats (Harlan laboratories) at 9 time points spanning development of the parotid gland, including embryonic day 18 (E18), E20, postnatal day 0 (P0; which is E22), P2, P5, P9, P15, P20, and P25.

### Ethics Statement

This study was carried out in strict accordance with the recommendations in the Guide for the Care and Use of Laboratory Animals of the National Institutes of Health. The protocol was approved by the Institutional Animal Care and Use Committee of the University of Louisville (Permit Number: 11059).

Timed pregnant females were used for the embryonic and early postnatal time points. Birth (P0) in this strain is typically on E22. All animals were euthanized by carbon dioxide inhalation or decapitation of embryos following IACUC-approved procedures. For animals older than P5 the parotid gland was removed, embedded in Tissue-Tek CRYO-OCT Compound (Fisher Scientific) and immediately frozen with a mixture of dry ice and 100% 2-methyl butane. For the embryonic and earlier postnatal time points, heads were divided along the sagittal plane, and each half was embedded upright. All tissue blocks were stored at -80°C. For cryosectioning, blocks were thawed to -30°C. Sections (7 μm) were taken with a Leica cryostat onto sterile chilled slides that had been treated with RNaseZap (Ambion), and immediately fixed in 70% ethanol. Xylenes and 100% ethanol were used to remove OCT before staining. For the heads, tissue was sectioned and stained with hematoxylin and eosin (H&E) until the parotid gland was located. All tissue sections were lightly stained with H&E to identify acinar cells based on the structure of the cells and local vascular landmarks, as validated by immunofluorescent staining of previous samples using anti-parotid secretory protein antibody. Stained sections were dehydrated by washing in 100% ethanol and xylenes before being used for microdissection.

Laser capture microdissection (LCM) was performed on an Arcturus PixCell *IIe LCM* System (Life Technologies/ Thermo Fisher Scientific) [27, 28]. Caps containing CapSure transfer film carrier were applied to the tissue and cells were adhered to the cap using laser pulses. The cap was then checked under the microscope to ensure that contaminating cells were removed. Only caps containing pure populations of the cells of interest were used in subsequent experiments.

### RNA isolation, microarrays, and qPCR arrays

RNA was isolated from the LCM caps using the RNaqueous micro kit (Ambion), per the manufacturer’s instructions. Briefly, once cells were isolated onto an LCM cap, lysis buffer was applied immediately. Tubes were then incubated in a heat block at 42°C for 30 min. The lysates were either processed immediately or stored at -80°C. Lysates from multiple caps of the same sample, taken on the same day were combined before proceeding with isolation. At least three independent biological samples (from separate litters) were used for isolation of total RNA at each of the 9 time points. Quantity and quality of the total RNA was assessed using a 2100 Bioanalyzer (Agilent). Samples with a RIN value of at least 7 were used.

For analysis of mRNA expression, the Whole Transcriptome-Ovation Pico RNA amplification system (NuGen Technologies Inc.) was used to prepare amplified cDNA from total RNA for 9 time points of the developing parotid acinar cells. The biotin-cDNA was hybridized to 27 separate rat genome 230 2.0 Affymetrix GeneChips, having 31,099 probe sets. Results for each chip were analyzed using standard Robust Multi-array Average (RMA) method for background correction, normalization and summary. Differential gene expression between time points (or between averaged stages of development) was determined by ANOVA or by using Linear Models for Microarray Data (LIMMA) software to test the false discovery rate (FDR)-adjusted significance of fits to several models (e.g., models with linear, quadratic, and cubic polynomials in time) [29]. Differentially expressed mRNAs were subsequently clustered using hierarchical clustering, and these clusters were analyzed for significant enrichment of biological processes (see description under Network Analysis below). Validation of the microarray data was performed using TaqMan Universal PCR Master Mix (Applied Biosystems; ABI) on an ABI 7500 Real-Time PCR System using TaqMan primers for the target mRNAs (*Psp*, *Xbp1*, and *Nupr1*), and for control *Rplp2* mRNA. Data were analyzed with Sequence Detection Software v1.4 normalizing to *Rplp2* expression between samples.

MicroRNA expression was measured by qRT-PCR at four time points during acinar differentiation: E20, P5, P15, and P25. A total of 372 primer pairs (miRCURY LNA, Exiqon) were used which amplify well annotated rodent microRNA sequences. Triplicate samples were run for each time point. Total RNA (1 ng) was used to synthesize cDNA (Exiqon’s Universal cDNA Synthesis Kit). The cDNA was then applied to microRNA Ready-to-Use PCR, Mouse&Rat panel I, V1.M (Exiqon) per the manufacturer’s instructions. Briefly, each 20 μl cDNA reaction was diluted 110x in nuclease free water and then combined 1:1 with 2x SYBR green master mix. The reactions were run on an ABI 7500 RT-PCR system. Missing replicates in the qRT-PCR data were imputed using the k-nearest neighbor algorithm, as described [30–32]

Expression values (CT values) for each array were normalized to the median expression by subtracting the median CT value for that array. Differentially expressed (DE) miRNAs across the four time-points were determined as described above for mRNAs.

### Metacore Analysis

Expression data for differentially expressed (DE) mRNAs were loaded into the knowledge-based program MetaCore (Thomson Reuters Inc., Carlsbad, CA). Statistically clustered sets of DE mRNAs were interrogated for enrichment of biological pathways using the using the Metacore Gene Ontology enrichment analysis algorithm, with the Affymetrix Rat Genome 230 array as the background dataset. These clustered sets were also analyzed for significant enrichment of transcription factors to understand the active regulatory processes. Also, in order to identify regulatory pathways related to differentiation, a network was constructed initially based on the profiles of transcription factor mRNA expression over time. For example, an early increase of a transcription factor mRNA coincident or followed by an increased mRNA of a known target gene would suggest a possible network interaction. Possible interactions (edges) needed to be consistent with Metacore knowledge-based interactions, or supported by the presence of an appropriate DNA-binding sequence in the target promoter. Interactions in initial networks were tested by transfections and ‘pruned’ as appropriate, and the network was expanded using Metacore's "expand" algorithm filtering for transcription regulatory interactions involving DE genes generated in this study. Targetscan [33–35] software was used to obtain predicted gene targets of DE microRNAs among the set of DE mRNAs. miRNAs predicted to target genes in the constructed network were added if their expression profiles had a statistically significant inverse correlation. Metacore's Pathway Map Creator was used for visualization of the derived network.

### Luciferase Reporter Transfections

Reporter assays were performed to experimentally test the predicted regulatory interactions (edges) of the network. Standard methods were used to clone the target gene promoters upstream of luciferase in pGL4.10 vector. Expression plasmids for selected transcription factors were from Open Biosystems (Huntsville, AL) or Thermo Scientific (Waltham, MA) or were cloned by RT-PCR of rat genomic DNA into pCDNA4 (S1 Table). Luciferase reporter plasmids were transiently transfected into the rat parotid gland derived ParC5 cell line or the closely related ParC10 [36, 37] cells (obtained from Dr. Quissell's laboratory) plated on 6-well tissue culture plates in DMEM/F12 media FBS and growth factors. Lipofectamine (Invitrogen) was used for luciferase promoter studies, following the manufacture’s guidelines. The *Mist1* transcription factor expression clone was in pcDNA4 vector, and the spliced *Xbp1* (*Xbp1*-S) expression vector was pFLAG.*Xbp1*p.CMV2 (Addgene; Cambridge, MA). In all experiments, pGL4.73-Renilla luciferase plasmid was co-transfected as an internal control for normalizing transfection efficiency. After 48 hours, cell extracts were prepared using Passive lysis buffer (Promega) and assayed for both firefly and Renilla luciferase activities using the Luciferase Assay and Renilla Luciferase Assay Systems from Promega with a Berthold Lumat LB9501 luminometer. The fold activation was calculated relative to cells transfected with the basal promoter-containing vectors alone. Each experiment was performed in triplicate, and repeated at least three times. Results are given as means ±SEM.

For miRNA transfections, miRNA mimics (Dharmacon, Lafayette, CO) were transfected at 10 pmols/well along with 100 ng of a 3'UTR containing plasmid into HEK293 cells using DharmaFeCT Duo (Dharmacon, Lafayette, CO). 3'UTRs were cloned down-stream of firefly luciferase cDNA in PGL4.25 using XbaI and FseI (S1 Table). Renilla luciferase plasmid (PGL4.73) was used as a transfection efficiency control. Empty PGL4.25 (no 3'UTR) was used as a normalization control vector and results were analyzed as in Jacobs et al., 2004 [38].

## Results

### Gene expression changes during acinar cell differentiation

**Fig 1:**
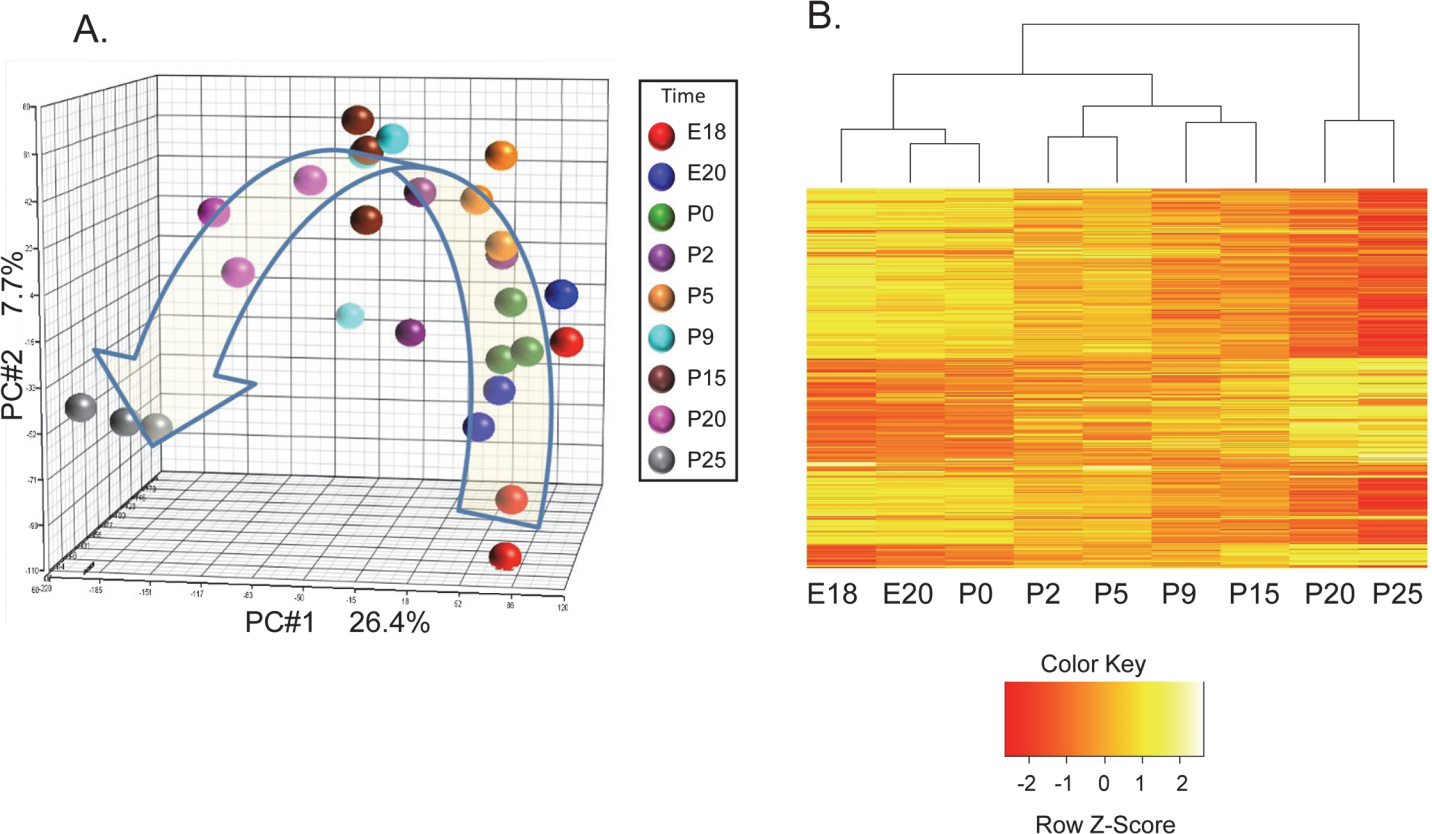
Hierarchical Clustering Divides Parotid Acinar Cell Differentiation into Stages. (A) Principal component analysis (PCA) of the 27 microarrays of RNA samples taken in triplicate at nine time-points across acinar cell differentiation. The first three principal components are plotted on a three dimensional graph. Samples are largely clustered based on the replicate time points. (B) Hierarchical clustering of differentially expressed mRNAs displayed as a heatmap suggests the presence of 4 stages. 2656 genes were identified as differentially expressed by one-way ANOVA (FDR p < 0.05). Image was generated using the heatmap.2 function in R and distance was calculated as dissimilarity = 1-r (correlation coefficient). Expression values are scaled (mean 0, std dev of 1) by rows.

A unique aspect of this study is the application of laser capture microdissection (LCM) to procure total RNA from populations of acinar cells across multiple time points. RNA from 9 time points from triplicate litters were collected across rat parotid gland development. These time points span pre-acinar terminal clusters, granule differentiation, and rapid maturation [13, 14]. Populations of acinar cells were isolated from parotid tissue sections without appreciable contamination from surrounding ducts and connective tissue, as indicated by hematoxylin and eosin stained LCM sections (S1 Fig). Principal component analysis of results from 27 microarrays showed appropriate grouping of the replicate samples, and indicates the major variability across the complete set of arrays is due to the different time points (Fig 1A). This supports the quality of the dataset. ANOVA of the entire dataset identified differential expression of 2656 mRNAs with FDR adjusted p-value of 0.05 or less. Hierarchical clustering of these 2656 mRNAs (Fig 1B) generated a dendrogram which suggests that the transcriptional profile entails four stages during parotid differentiation, e.g., embryonic (E18, E20, and P0), early postnatal (P2, and P5), middle postnatal (P9, and P15), and late postnatal (P20, and P25). The heat map indicates that relatively little change occurs in the prenatal period, but a large number of expression changes occur just after birth, and another large number of expression changes occur late in differentiation. This is consistent with previous reports based on expression of individual proteins suggesting that terminal differentiation occurs in several stages [13, 14]. In our study, direct comparison between adjacent stages identifies hundreds of significant changes of mRNA expression (Table 1). Similar statistical comparison between ages within a stage yields far fewer differentially expressed mRNAs (data not shown). In order to validate the microarray data, a subset of genes was measured by qPCR. The expression patterns for 4 specific mRNAs (*Psp*, *Xbp1*, and *Nupr1*) were tested by TaqMan qPCR with the same total RNA samples, and yielded results similar to that of the microarrays (S2 Fig).

**Table 1:** Gene Expression Comparisons between Developmental Stages.

Stage Comparisons	# of Differentially Expressed mRNAs
Stage 1 – Stage 2	604
Stage 2 – Stage 3	124
Stage 3 – Stage 4	992
Stage 1 – Stage 4	3506

### Clustering of Differentially Expressed mRNAs

**Fig 2:**
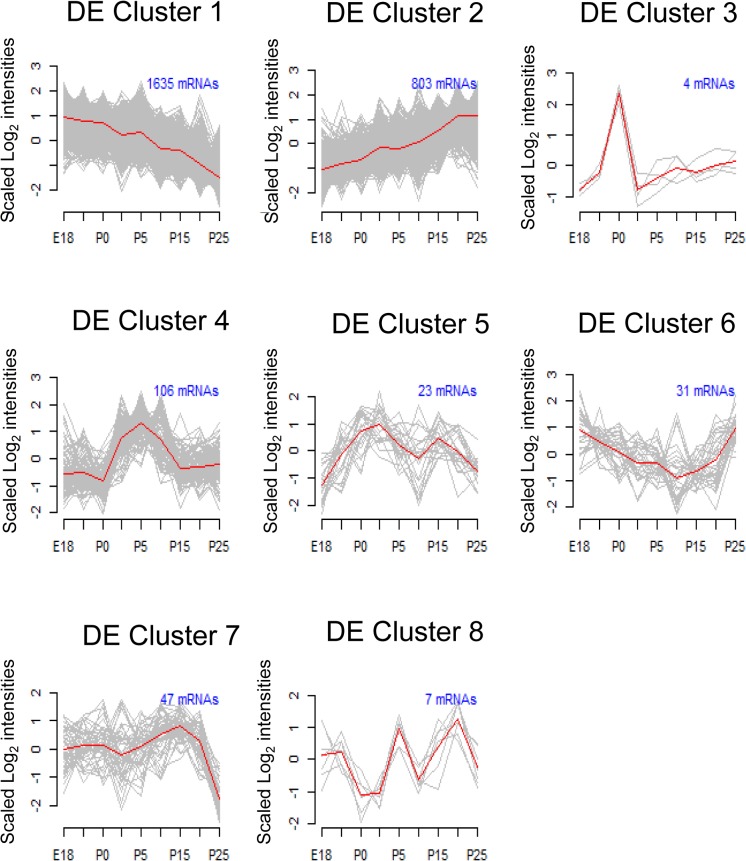
Profiles of Differentially Expressed mRNAs during Differentiation. The time course microarray data were analyzed to identify differentially expressed mRNAs, which were clustered based on statistically significant similarities in the expression profiles. For visualization, the expression data for each gene was scaled to a mean of zero and standard deviation of 1 before plotting. The red line traces the average expression for the cluster.

All differentially expressed (DE) mRNAs were submitted to cluster analysis (Fig 2). Most mRNAs fell into progressively decreasing (DE cluster #1; 1635 mRNAs) or progressively increasing (DE cluster #2; 803 mRNAs) clusters. Metacore Gene Ontology (GO) enrichment analysis indicated that DE cluster #1 is significantly enriched for genes related to mitosis and the cell cycle. The top 6 processes and the top 4 pathway maps each involve cell cycle. All the significantly enriched processes and pathway maps are shown in supplemental S2 Table. Progressively decreasing genes in DE cluster #1 include *PCNA*, cyclinD1/2, and cyclinB2, which are directly involved in regulation of cell cycle. In addition, *Mcm 3*, *4*, *5*, *6*, *7*, *and 10*, which are essential for the initiation of DNA synthesis each decrease across development. The observed gene enrichments are consistent with the parotid cells transitioning from actively dividing to terminally differentiated. In addition, this GO analysis suggested decreased expression of three signaling pathways which stimulate epithelial–mesenchymal transition (EMT) (Notch, TGFβ, hypoxia), consistent with the formation of the epithelium of the acini (S2 Table).

DE cluster #2 (genes with increasing expression) showed a completely different set of enriched processes and pathways. This cluster is enriched in GO terms related to ion transport, lacrimal gland function, and the endoplasmic reticulum, such as ER-nucleus signaling and the unfolded protein response (S3 Table). As anticipated, this cluster includes increased expression of mRNAs related to parotid acinar cell function, e.g., secreted proteins (amylase, parotid secretory protein (*Psp*, *Bpifa2e*), lysozyme, *Lbp*, and lactoperoxidase) and multiple transmembrane transporters important to parotid function (Aquaporin 5, *Orai1*, *Atp1a1*, *Nkcc1*, *Slc9a1*, *Slc41a2*, *Slc26a6*, *Slc39a2*, *Slc7a8*, *Kcc2*, *P2rx4*). NKCC1 (Na+/K+/2Cl−cotransporter) protein is expressed specifically in the basolateral membrane of parotid acinar cells and knocking out its gene impairs salivation [39]. SLC9A1/Nhe1 (a Na+/H+ exchanger) is a major regulator of pH in parotid acinar cells [40]. This identifies genes related to parotid acinar cell function which show a progressive increase across differentiation. In order to understand factors important to gene regulation, the 803 genes in this cluster were tested for significant enrichment of targets of any transcription factors in the Metacore database. DE cluster #2 is significantly enriched in targets of only 3 transcription factors, *Mist1*, *Xbp1*, and glucocorticoid receptor (S4 Table). Both *Mist1* and *Xbp1* are also members of this cluster. The increases seen in the expression of mRNAs in this cluster support the utility of this dataset for modeling of parotid differentiation.

**Fig 3:**
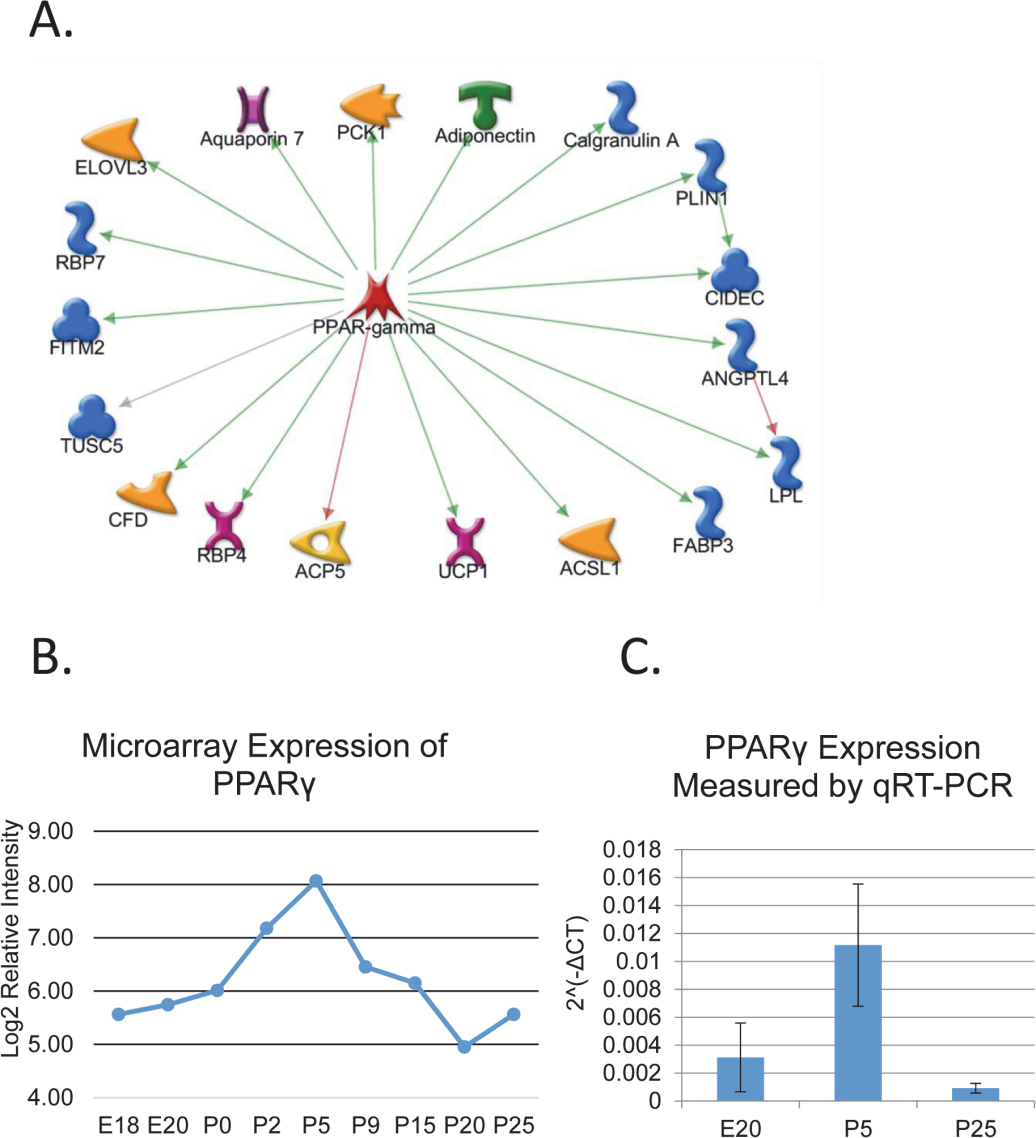
Transient Activation of *Pparg* during Parotid Acinar Cell Differentiation. (A) Network showing transcription factor *Pparg* and known downstream target genes found in DE Cluster 4 (Fig 2). DE Cluster 4 contains 106 genes (including *Pparg*) with a unique expression pattern; higher expression only in stages 2 and 3. The Metacore knowledge-base identifies 18 of these as *Pparg* target genes. A green arrow indicates activation of transcription while red arrow indicates inhibition. A grey line means the interaction is uncharacterized. Although a red arrow connects *Pparg* and *ACP5*, some publications list the interaction as activating [67, 68] indicating it could be context dependent. (B) Log2 expression of *Pparg* from microarray data. (C) qPCR data confirming the expression profile of *Pparg*. RNA samples from independent animals were collected at three time points (E20, P5, and P25). Expression was normalized to *Arbp*, and data showed significant change in expression by ANOVA. n = 3.

Interestingly, an additional cluster containing 106 differentially expressed mRNAs was identified having increased expression only in the mid-development stages (DE Cluster 4, Fig 2). This cluster is highly enriched in genes involved in adipocyte differentiation and lipid metabolism, and includes transcription factor *Pparg* (S5 Table). Transcription factor target analysis revealed a hub containing *Pparg* and 18 known downstream targets (Fig 3A). qPCR of independent samples confirms transient up-regulation of *Pparg* mRNA expression around postnatal day 5 (Fig 3C). This suggests a novel dimension of parotid differentiation involving *Pparg* regulation, yet. this agrees with the previous work of Mukunyadzi et al which found that adult parotid acinar cells do not express *Pparg* [41].Other statistical clusters derived from the DE mRNAs (Fig 2) had no significant GO enrichment.

### Quadratic model mRNA clusters

**Fig 4:**
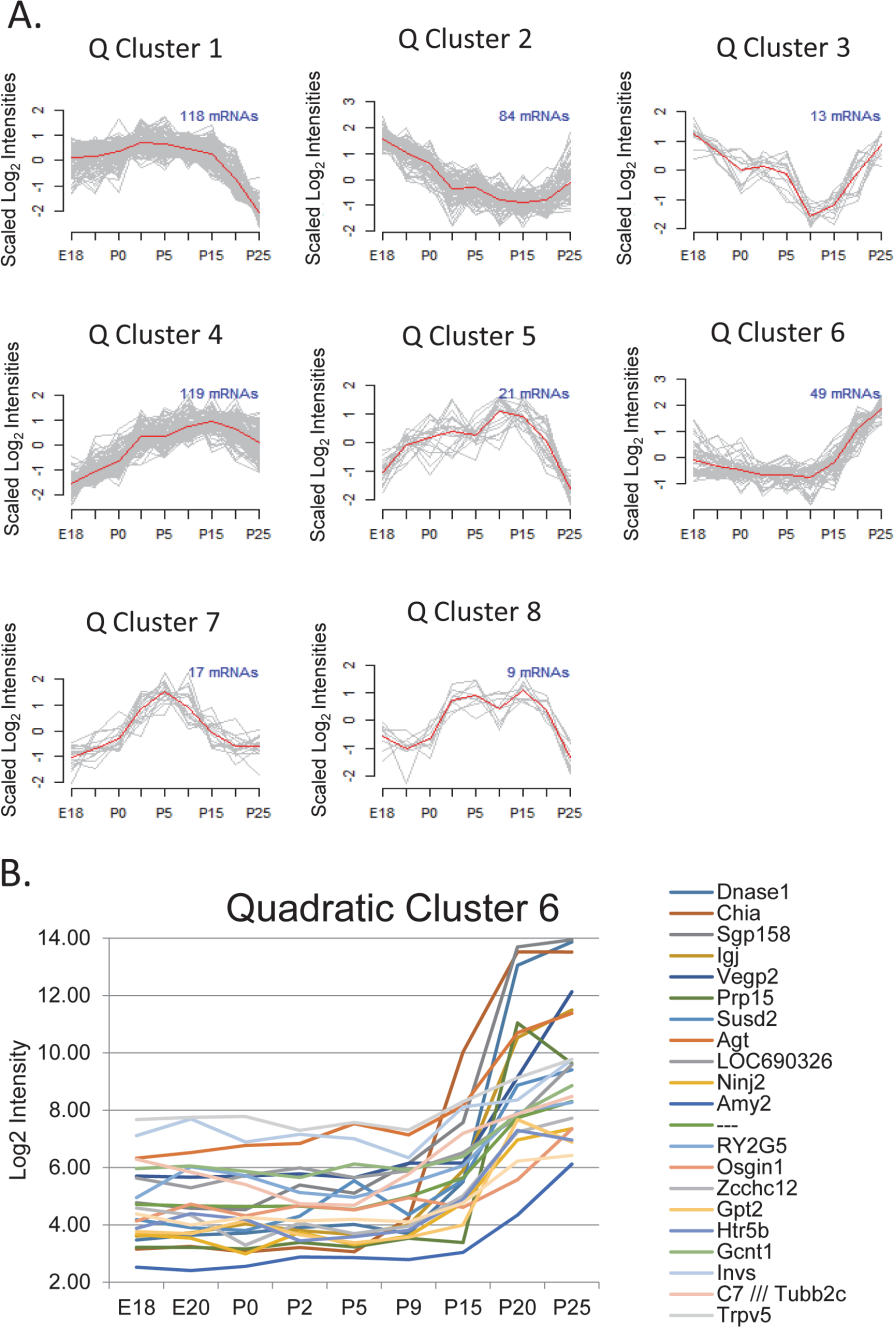
mRNAs with a Quadratic Expression Pattern. Quadratic regression reveals late activation of acinar cell specific genes. (A) Quadratic regression analysis identified 430 genes having a significant quadratic trend (FDR < 0.05), which were clustered into eight patterns. For visualization, the expression data for each gene was scaled to a mean of zero and standard deviation of 1 before plotting. The red line traces the average expression for the cluster. (B) Log2 plot of Quadratic Cluster 6 members with at least a 4-fold expression difference between P25 and E18. This shows late up-regulation of several genes known to produce salivary proteins (i.e. *DNase I*, *Chitinase*, *Prp15*, *Sgp158/Prr21*).

To characterize gene expression patterns further, the microarray results were submitted to regression analysis to identify mRNAs significantly fitting either a quadratic or a cubic polynomial model (based on FDR-adjusted p values). Few (18 mRNAs) fit the cubic patterns and they were not investigated further (S4 Fig). However, after FDR correction, 430 mRNAs showed a significant match to quadratic trend models and were used for cluster analysis (Fig 4A and S3 Fig). The largest, Quadratic cluster #1, contained 118 mRNAs with high expression through most of differentiation and a strong decrease during the last stage. GO enrichment analysis identifies extracellular matrix and cell adhesion as relevant processes (S6 Table). This cluster contains collagens (*Col1a1*, *Col5a2*, *Col1a2*, and Collagen XIV) as well as other ECM proteins such as fibrillin-1, Tenascin C, and Laminin alpha 5. Tenascin C is typically expressed during organogenesis but not in adult tissues [42]. Apparently, these genes are involved in differentiation of the ECM and organogenesis, and decrease rapidly as the parotid gland completes development. Quadratic cluster #6 identifies an expression pattern which is reciprocal to #1; 49 mRNAs exhibited low expression throughout early and mid-development, but a strong increase was observed in the last stage (Fig 4B). Genes in this cluster include *Chia*, DNase1 and proline-rich protein 15 (*Prp15*) each of which are secreted salivary proteins. Fig 4B demonstrates that the full secretory repertoire is not established until the last stage of acinar cell differentiation. A separate cluster (Quadratic cluster #7, Fig 4A) independently identified a subset of those genes in DE cluster #4. This cluster also contains *Pparg* and 4 of its target genes as described above, and as indicated by the GO enrichment analysis (S7 Table). Overall, cluster analyses of the results from the 27 microarrays supports the utility of this dataset by identifying patterns of mRNA expression which are consistent with our broad understanding of parotid development. Cluster analyses shows large changes in important salivary proteins even late in differentiation (Fig 4). In addition, this analysis suggests a novel role for *Pparg* or closely related factors during the middle stages of parotid gland development.

### MicroRNA expression changes during acinar cell differentiation

**Fig 5:**
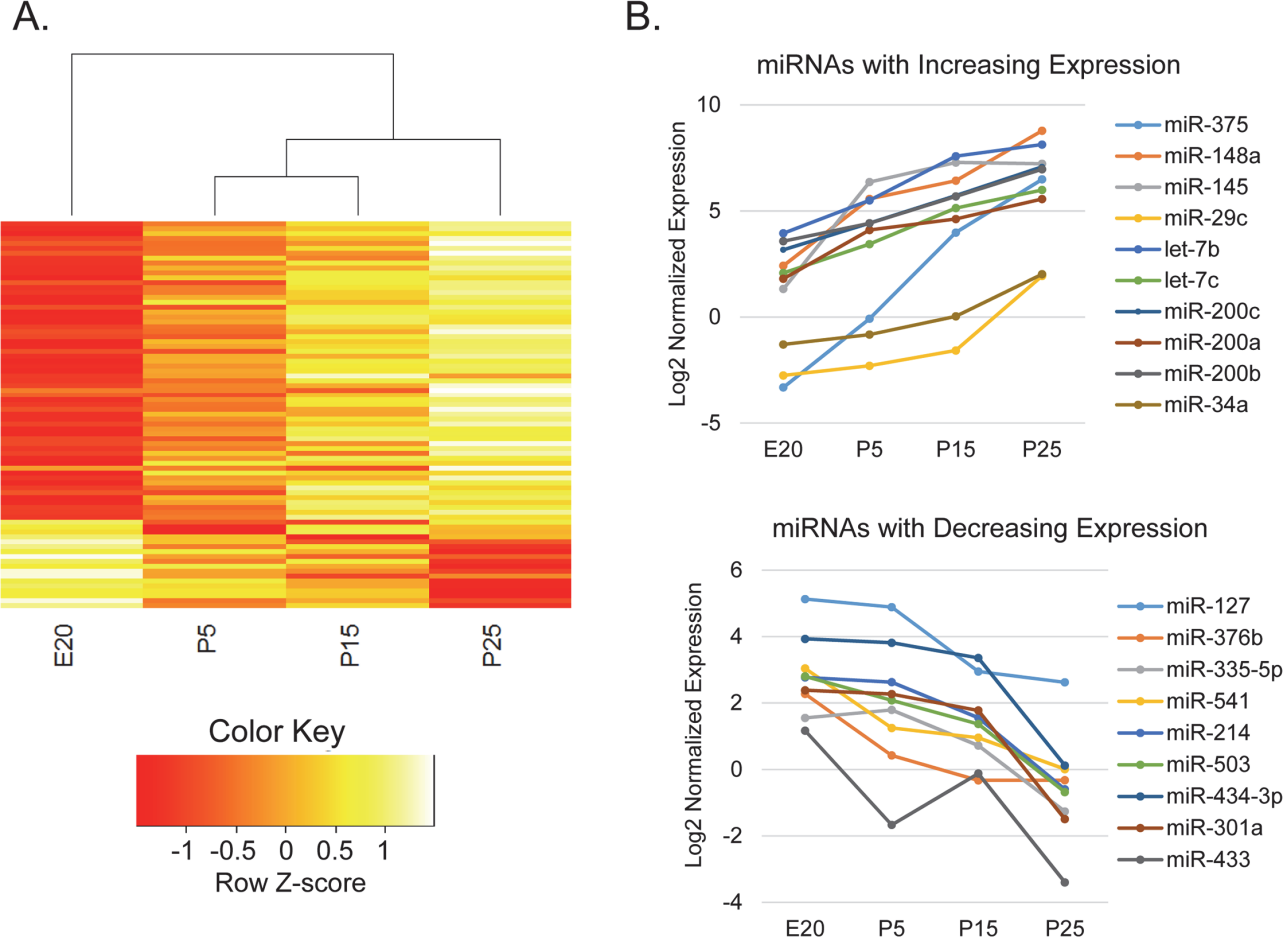
miRNA Expression Changes in Parotid Acinar Cell Differentiation. Significant expression changes in miRNAs are seen across parotid acinar cell differentiation. (A) Heatmap of 79 miRNAs that are differentially expressed during parotid acinar differentiation, by one-way ANOVA (p < 0.05). Heatmap was generated as in Fig 1. (B) Log2 plots of miRNAs with large significant expression changes.

In order to derive a regulatory network relevant to parotid acinar cell differentiation, it is essential to include changes in microRNA expression. The relative expression of 372 microRNAs was measured by qPCR (with the Exiqon mouse+rat miRNA panel) in triplicate LCM samples at each of four time points across acinar cell terminal differentiation (E20, P5, P15, and P25). The time points were chosen based on the observation of 4 stages with the mRNA microarrays, described above (Fig 1B). qRT-PCR detected 271 microRNAs repeatably. Analysis by one-way ANOVA identified 79 miRNAs exhibiting significant differential expression (Fig 5A). Subsequent t-tests between time points (Table 2) identified 64 miRNAs with differential expression between the first (E20) and last (P25) time points. These 64 miRNAs encompass all significantly changing miRNAs identified by comparing other pairs of time points. Of these, 52 miRNAs increased in expression and 12 decreased across acinar differentiation. Within the limitations of 4 time points, most miRNAs appeared to change expression linearly, with none being transiently activated or depleted. Linear regression analysis identified two clusters, either progressively increasing or decreasing (S5 Fig). No miRNAs had a significant quadratic expression pattern. Plots for miRNAs with fivefold or greater expression change between E20 and P25 are shown in Fig 5B. The miRNA with the largest expression change, miR-375, increased more than 800-fold. This miRNA has been identified as being expressed in adult salivary glands. Its target gene, *Plag1*, is a transcription factor which stimulates proliferation, and the increase of miR-375 may therefore contribute to decreased proliferation during differentiation. *Plag1* is a proto-oncogene that is often up-regulated in pleomorphic adenomas of the salivary glands, a cancer that makes up 70% of parotid tumors. Expression of miR-375 is often down-regulated in salivary cancer cells [43].

**Table 2:** MicroRNA Expression Changes between Time Points.

Time Point Comparisons	# of Differentially Expressed miRNAs
E20 vs. P5	1
E20 vs. P15	16
E20 vs. P25	64
P5 vs. P15	0
P5 vs. P25	7
P15 vs. P25	0

### Transcription factor and microRNA regulatory network modeling

mRNA data were integrated with the microRNA array data to identify potential targets which could have biological significance during differentiation. Briefly, potential targets of the 64 DE miRNAs (identified by ANOVA and t-test) were generated in silico from the collection of 2656 mRNAs that changed significantly during differentiation. The computer algorithm Targetscan [44] was used to compile a list of predicted targets; a miRNA core sequence in a target mRNA 3’UTR was essential for inclusion in the model. Differentially expressed mRNAs were considered as targets of interest, leading to the identification of 5184 potential target sites in 851 unique mRNAs (S8 Table). Interestingly, these genes are significantly enriched in DE cluster #1, and deficient in DE cluster #2 (Fig 2) (Fisher's exact test p-value < 0.05) (S9 Table) indicating that miRNAs may have an important impact on overall gene expression trends during differentiation. Of the decreasing genes in DE cluster #1, 32% are predicted to be direct targets of microRNAs which increased expression.

**Fig 6:**
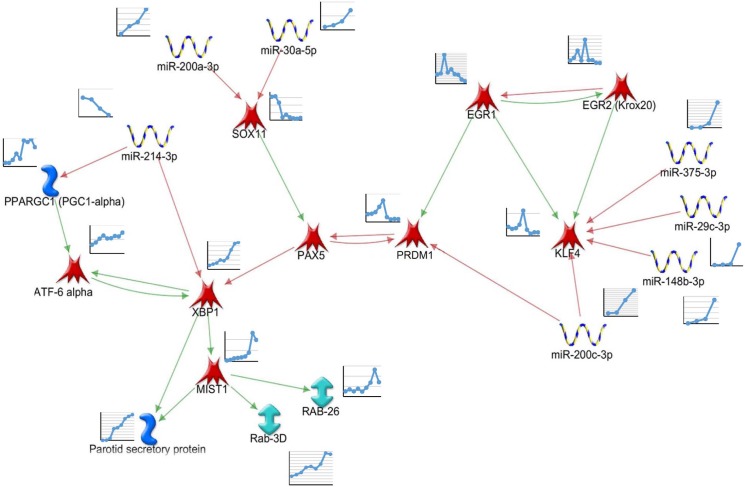
Regulatory Network Driving Markers of Acinar Cell Differentiation. Genes and miRNAs were analyzed for either consistent or inverse expression changes during the time points measured. Selected genes were analyzed by Network Analysis (Metacore) to identify gene regulatory interactions, focusing particularly on parotid specific markers of terminal differentiation. A green line in the network indicates transcriptional activation, while a red line indicates inhibition. MicroRNA target genes were identified through Metacore and/or Targetscan and subsequently tested for inverse correlation.

In order to identify regulatory pathways related to differentiation, all DE transcription factor mRNAs, as well as predicted targeting miRNAs were used to generate putative regulatory networks. The network was initially based on the profiles of transcription factor mRNA expression over time, consistent with Metacore knowledge-based interactions, including potential links to markers of terminal differentiation (*Psp*, amylase, RABs). Nodes (mRNAs or miRNAs) or edges (predicted interactions) were only included where the predicted interactions matched the observed changes (increases or decreases) of the transcription factor target gene mRNA over time. miRNAs predicted to target mRNAs in the constructed network were only included if their expression profiles had a statistically significant inverse correlation. Initial networks were expanded using Metacore's "expand" algorithm, filtering for transcription regulatory interactions involving DE genes generated in this study. The derived network based on the observed changes of gene expression is depicted in Fig 6, and was further investigated by transfection studies experimentally testing individual edges.

The hypothetical network (Fig 6) suggests that expression of *Egr1* early in development maintains expression of *Klf4 [45].* Similarly to the parotid, *Egr1* is highly expressed in hematopoietic stem cells and decreases on differentiation [46]. *Klf4* is involved in stem cell maintenance and inhibits terminal differentiation [47]. As development proceeds, the observed increases of miR-29c, miR-375, miR-148, and miR-200c may drive the observed decreased expression of *Klf4* mRNA. *Sox11* is initially strongly expressed, and is an activator of the *Pax5* gene, which is an inhibitor of *Xbp1* transcription factor gene expression [48]. Increasing expression of miR-200a and miR-30a may combine to repress expression of *Sox11*, thereby decreasing stimulation of *Pax5*. *Prdm1* (*Blimp1*) mRNA increases transiently during mid-differentiation, which may inhibit *Pax5*. The *Prdm1*-*Pax5*-*Xbp1* genes are reported to form a genetic switch which regulates the timing of differentiation of antibody secreting plasma B cells [49, 50]. This genetic switch has not previously been seen in parotid differentiation, and may contribute to the observed increase of *Xbp1* mRNA.

The observed decrease of miR-214 which may target *Xbp1* mRNA, combined with the positive feedback loop between *Xbp1* and *Atf6* alpha [51, 52], would help maintain the observed elevated expression of *Xbp1*. The observed increases in expression of *Xbp1* and *Mist1* likely contribute to stimulating markers of terminal differentiation in the parotid gland such as salivary proteins and secretion-related proteins (as in Quad Cluster #6, above) and structural organization of the acinar cell [15].

**Fig 7:**
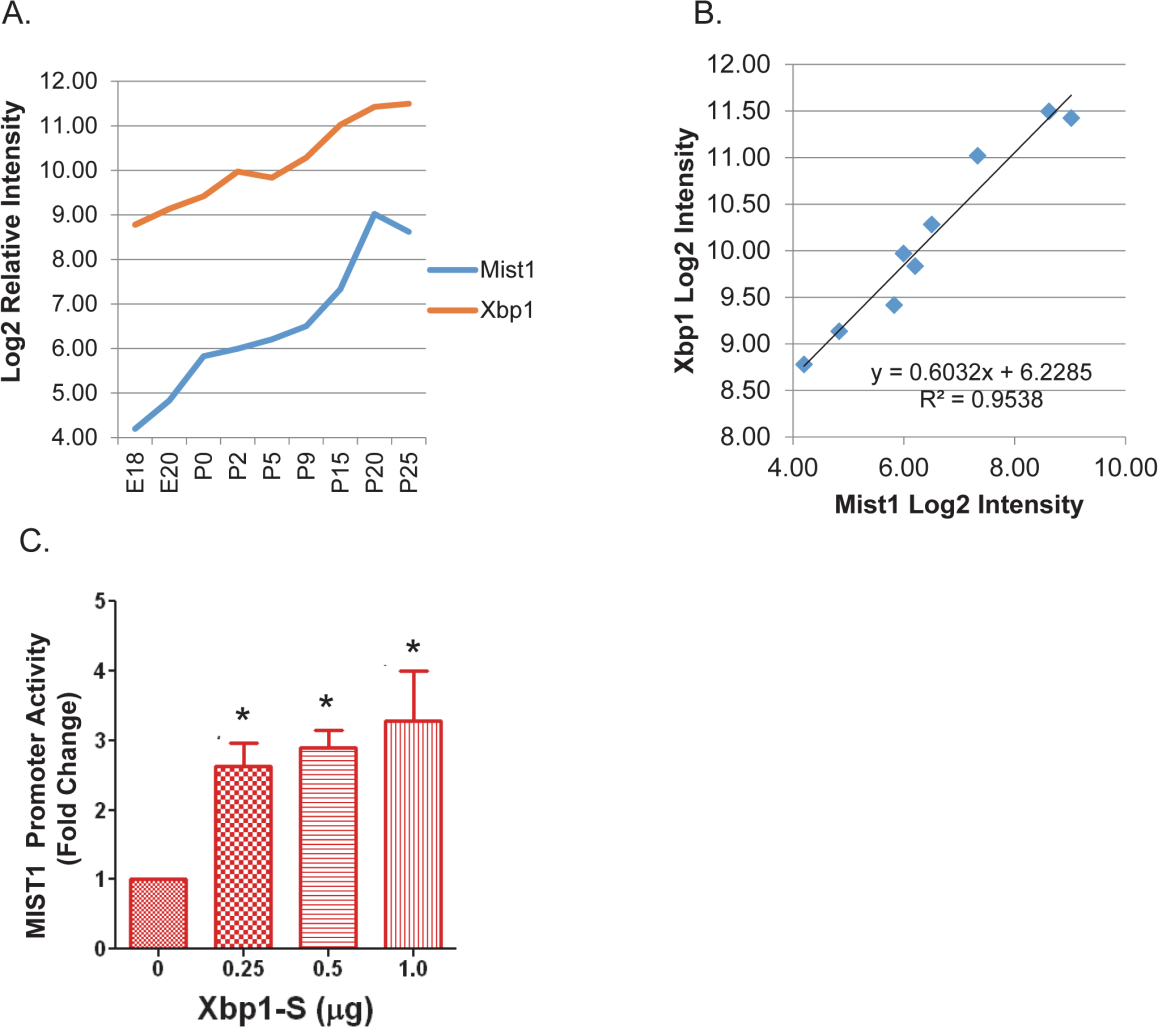
*Xbp1* Regulates *Mist1* Expression during Parotid Differentiation. (A) Log2 plot of microarray data for *Xbp1* and *Mist1*. (B) Expression of *Xbp1* and *Mist1* is highly correlated across parotid differentiation. Plot of Log2 *Xbp1* vs. Log2 *Mist1* shows a linear trend with R^2^ = 0.9538. (C) Luciferase assay shows activation of *Mist1* promoter by *Xbp1* in ParC5 cells. Increasing amount of *Xbp1*-S (spliced *Xbp1*) cDNA/well (0.25 μg, 0.5 μg, and 1 μg) were co-transfected with a luciferase expression plasmid driven by a *Mist1* promoter. Significant increase in luciferase expression was observed for all concentrations of *Xbp1*-S (p = 0.017, p = 0.01, and p = 0.05 respectively) (n = 3).

Eight of the proposed regulatory interactions (edges) of this network were directly tested by transfection experiments. According to the network, *Xbp1* is directly up-stream of *Mist1* (Bhlha15) [53] [54]. *Mist1* and *Xbp1* in vivo expression increases 21-fold and 6.5-fold respectively between the earliest and latest time points (Fig 7A). Their expression pattern was significantly correlated across the time points measured (correlation coefficient = 0.97, p-value = 1.4*10^–5^) (Fig 7B). Transfections of a *Mist1* promoter (-500 - +15) luciferase construct into immortalized rat parotid acinar cells (ParC5 cell line) confirmed direct activation of the *Mist1* promoter by the spliced (activated) form of *Xbp1* (Fig 7C). This experimentally supports the predicted interaction in this network.

**Fig 8:**
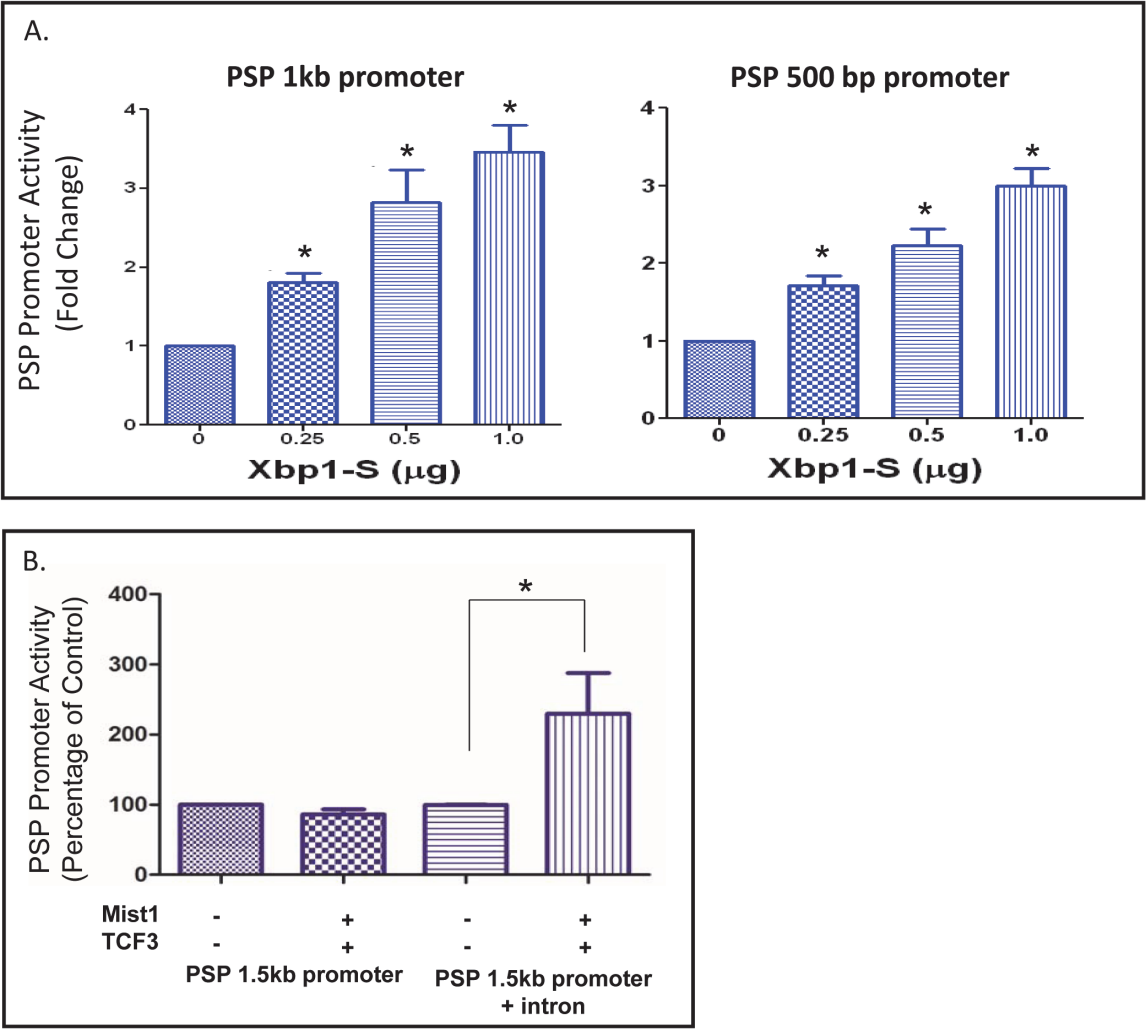
*Psp* is Directly Regulated by both *Xbp1* and *Mist1*. (A) *Xbp1* activates the *Psp* promoter. Increasing amounts of *Xbp1*-S cDNA was co-transfected into ParC5 cells along with a luciferase expression plasmid driven by either a 500 bp or 1 kb fragment of the *Psp* promoter region. Analysis was performed by t-test. Expression of luciferase driven by 1 kb *Psp* promoter increases significantly upon increasing transfection of *Xbp1*-S (p = 0.007, 0.02, and 0.005 respectively). The same is seen with the 500bp *Psp* promoter (p = 0.01, 0.01, and 0.003 respectively) (n = 3). (B) *Mist1* activates the *Psp* promoter through interactions with intronic sequences. Luciferase expression was driven by either a 1.5 kb fragment of the *Psp* promoter or the 1.5 kb fragment along with 1320 bp of intronic sequence flanking exon 3 that contains two E-boxes. Promoter plasmids were co-transfected with *Mist1* and *Tcf3* cDNA expression plasmids. Analysis was performed by t-test (p = 0.02) (n = 4).

Since our goal is to suggest a regulatory network relevant to parotid differentiation, an important consideration was to anchor the predicted network to key parotid-specific proteins. Little is known regarding which transcription factors regulate major salivary proteins such as *Psp* or amylase. *Psp* expression increases dramatically during acinar differentiation, particularly after birth. The tissue specific promoter region of this gene has been identified [55], but the transcription factors regulating its expression remain unidentified. Two CCACG boxes were identified -36 and -48 bp upstream of the transcription start site (S6 Fig). This sequence is part of the ER stress response element (ESRE) and is a consensus sequence for *Xbp1* binding [51]. The spliced form of *Xbp1* (*Xbp1*-S) activated the 500 bp and 1kb *Psp* promoter in luciferase transfection experiments in ParC5 cells (Fig 8A). This supports that edge within the network, and indicates that *Xbp1* may directly contribute to the observed increase of *Psp* mRNA expression during development.

Regulation of the *Psp* gene by the *Mist1* transcription factor was also investigated. Co-transfection of *Mist1* cDNA did not lead to activation of the *Psp* 500 bp promoter (not shown), nor did it activate a 1.5 kbp *Psp* promoter construct even in the presence of the *Mist1* dimerization partner *Tcf3*/E2A [56](Fig 8B). Sequence analysis indicated the presence of two E-box sequences (CAGCTG) flanking exon 3 in the rat *Psp* gene. Since these are *Mist1* consensus binding sites, a 1320 bp rat DNA fragment from the end of exon 2 through the start of exon 4 was cloned (S1 Table) into the 1.5 kbp *Psp* promoter-luciferase construct. This promoter+exon construct was activated 2.3-fold by co-transfection with *Mist1* and *Tcf3* cDNAs (p = 0.0227), whereas the 1.5 kbp *Psp* promoter was not activated (Fig 8B). Neither *Tcf3* nor *Mist1* alone significantly activated the *Psp* reporter construct (not shown). *Tcf3* was included in these experiments as a heterodimerization partner with *Mist1*. Since *Tcf3* mRNA was not differentially expressed (p = 0.25), it is not included in the proposed regulatory network, however, it is constitutively expressed throughout acinar cell differentiation. While *Tcf3* is typically present in most cell types, it apparently has inadequate levels in the ParC5 cells used for transfections. Overall, these experiments indicate that the increase of *Mist1* expression during acinar differentiation contributes to the increase of *Psp* gene expression through binding sites flanking exon 3.

**Fig 9:**
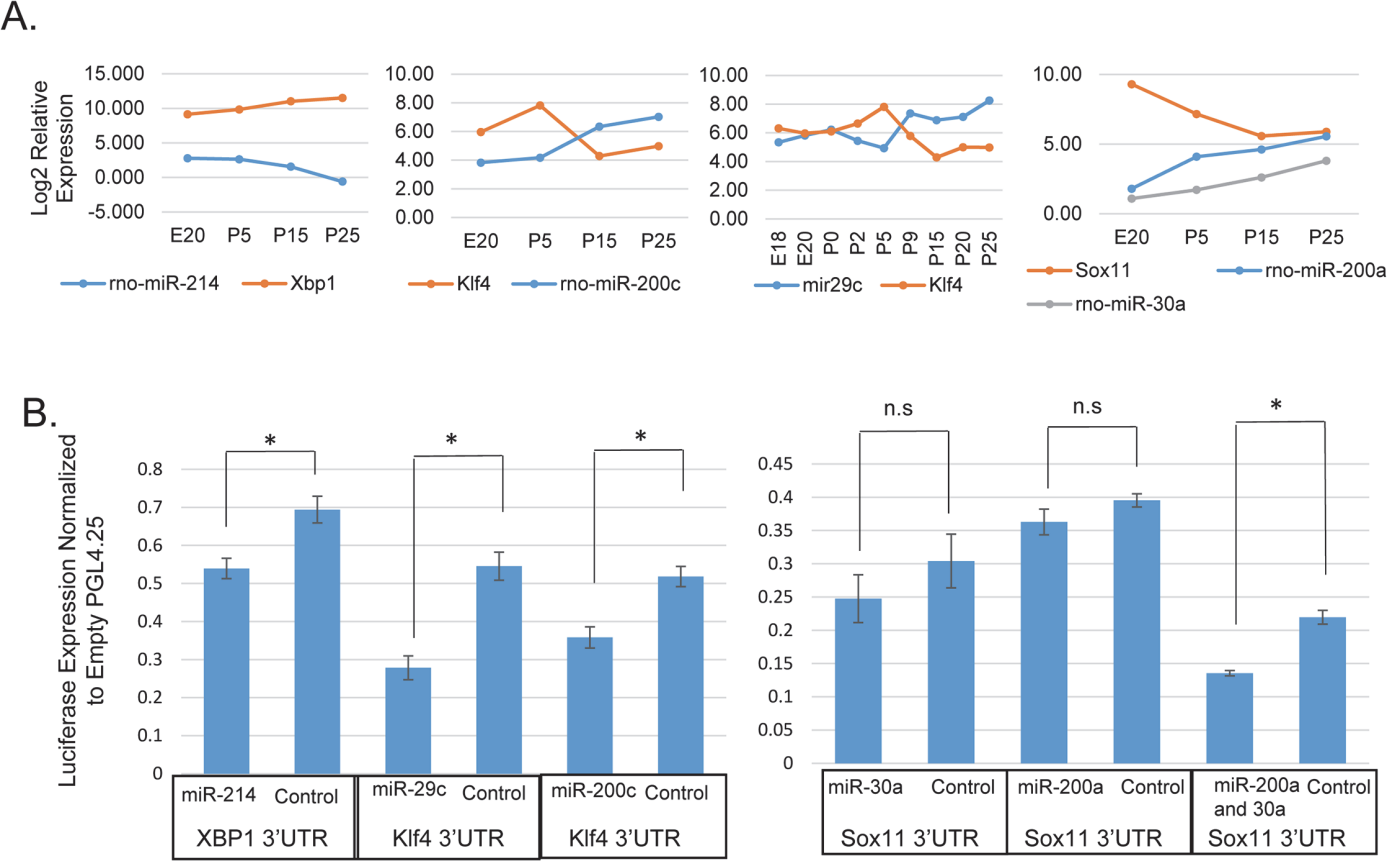
miRNA Target Genes Involved in a Regulatory Network. (A) Log2 plots of microarray and qPCR expression profiles for miRNAs and their predicted target genes: miR-214, and *Xbp1*; miR-29c, miR-200c and *Klf4*; and miR-30a, miR-200a, and *Sox11*. (B) miRNAs repress expression of their target genes as shown by luciferase assay. 3'UTRs were cloned downstream of luciferase and co-transfected along with a miRNA mimic. Analysis was done by two-sample t-test as in Jacobs et al. [38] comparing the targeting miRNA with a control miRNA. (n = 3 for miR-29c, miR-200a, miR-200c, and miR-200a/30a) (n = 4 for miR-30a and miR-214) (p = 0.014, 0.012, 0.025, 0.28, 0.23, and 0.017 respectively).

Seven miRNAs are included in the network through predicted target sites in five of the transcription factor mRNAs (Fig 6). Four miRNAs, which increased expression late in differentiation, are predicted to target *Klf4* mRNA. This includes miR-29c, which has been shown to regulate *Klf4* expression in breast cancer cells. Loss of miR-29c in those cells results in dedifferentiation due to *Klf4*, leading to a population of stem-like cells [57]. Fig 9A shows the Log2 relative expression of miR-29c during acinar differentiation compared to that of *Klf4* mRNA. *Klf4* does not decrease in expression until late in postnatal development when miR-29c increases. Expression of these two genes are negatively correlated across acinar differentiation (Pearson’s r = -0.79; p = 0.011). In order to test potential miRNA target genes, HEK293 cells were co-transfected with a miRNA mimic and a luciferase expressing plasmid containing a 3'UTR of interest. Transfection experiments confirmed repression of rat *Klf4* by miR-29c (Fig 9B). Several other miRNAs are predicted to target *Klf4*, among them miR-200c, which has relatively low expression in the embryo and early postnatal gland but significantly increased in expression by P15. It has been shown that *Klf4* is targeted by miR-200c [57, 58], and luciferase assays confirmed this interaction in a parotid cell line (Fig 9B).

*Xbp1* mRNA increased expression 6.5-fold across differentiation, while its targeting miR-214 decreased more than 10-fold (Fig 9A). *Xbp1* 3'UTR contains a known miR-214 binding site in humans [59]. Although this sequence is not conserved in rats (S7 Fig), the rat 3'UTR contains an alternate miR-214 predicted binding site, which was cloned into a reporter, and co-transfection experiments demonstrate repression of rat *Xbp1* by miR-214 (Fig 9B).

*Sox11* expression decreased significantly after birth, and it is predicted to be targeted by several differentially expressed miRNAs. Assays with miR-200a, and miR-30a did not show any repression of the reporter, however, a construct containing binding sites for both miR-200a and miR-30a was repressed when co-transfected with both miRNAs (Fig 9B), indicating that these miRNAs are acting cooperatively to repress expression. Taken together, these miRNA transfection experiments provide experimental support for five of the edges in the proposed network.

## Discussion

Terminal differentiation involves enduring changes in the gene expression patterns of a cell. We have defined profiles of expression of mRNAs and microRNAs across the month-long process of parotid acinar cell differentiation. Based on these profiles, we have generated a regulatory network which suggests transitions in gene expression driving parotid differentiation. The focus on acinar cell differentiation is supported by the use of laser-capture microdissection to isolate developing acinar cells at multiple time points. Incorporating time as a variable in this study allows comparison of the profile of expression of a transcription factor mRNA with the profile of a putative regulated gene. Co-variance supports inclusion of the transcription factor and its target gene in the regulatory network. Similarly, microRNAs were only included in the network when the profile of expression of the microRNA was inversely related to that of the target mRNA. This ensures that the network has both knowledge-based and expression level support which strengthens the results. This putative network provides a context for changes in transcription factors which regulate differentiation. The network identifies two main branches; initial expression of stemness factors (*Sox11*, *Klf4*, *and EGR1*, *none of which have been describe before in parotid differentiation*) which inhibit differentiation, and subsequent switch to an *Xbp1* pathway which drives and maintains markers of terminal differentiation. Our network suggests that Klf4 is initially regulated by Egr1 and, remarkably, subsequently repressed by 4 different miRNAs which increase strongly in the late stages. We demonstrate that either miR-200c or miR-29c can down-regulate the expression of *Klf4*. By affecting its expression, these miRNAs could be important drivers of terminal differentiation. This is one example of the broad observation that miRNAs have extensive roles driving parotid differentiation. Remarkably, 52 significantly increasing miRNAs target 524 significantly decreasing mRNAs, potentially contributing to direct regulation of 32% of the DE cluster #1 mRNAs.

*Sox11* expression was elevated in embryonic stage acinar cells, decreasing dramatically immediately after birth apparently due to concerted action by both miR-200a and miR-30a. *Sox11* is important in neurogenesis and involved in stem cell survival [60], and its down-stream factor, Tead2, is involved in maintaining ES cell identity and inhibiting differentiation [61]. In our mRNA profiles, *Tead2* directly parallels *Sox11* expression across parotid differentiation. Hence, repressing *Sox11* promotes differentiation and also diminishes a stemness program. *Sox11* directly activates transcription of the *Pax5* gene [48]. *Sox11* is not expressed in normal lymphoid progenitor cells, however, in mantle cell lymphoma tumors it activates *Pax5* thereby blocking differentiation [48]. While *Pax5* probes were not present in the microarray used in the current experiments, we infer that its expression decreases downstream of *Sox11* expression changes. Pax5 is a transcriptional inhibitor of *Xbp1*, and decreasing its expression would contribute to *Xbp1* activation. The *Prdm1*-*Pax5*-*Xbp1* genetic switch is well characterized in differentiating immune plasma cells [49, 50], and we suggest is active in parotid acinar cells, with the additional regulation by *Sox11*.

Parotid acinar expression of *Xbp1* is apparently maintained low in the embryo by dual repression entailing both direct repression by miR-214, and indirectly by *Sox11* activating the Pax5 repressor. Studies of *Xbp1* knockout mice have underscored the requirement for this transcription factor in pancreatic acinar development [24]. Pancreatic acinar cells of *Xbp1* null mice have poorly organized and sparse ER, virtually no secretory granules and pancreatic amylase is almost undetectable. In contrast, the submandibular salivary gland is less profoundly affected showing abundant secretory granules (although smaller), and only a 50% reduction of salivary amylase [24]. While the parotid gland in *Xbp1*-null mice was not described, based on the proposed regulatory network we would predict a dramatic impairment of parotid differentiation, more similar to the pancreas. Xbp1 and Mist1 likely work as 'scaling' factors, a concept developed by Mills et al. [62] which contributes to quantitative differentiation.

Downstream of *Xbp1*, the serous exocrine specific transcription factor *Mist1* is up-regulated. We confirm direct regulation of the *Mist1* promoter by *Xbp1* in a parotid cell line (Fig 7C). Several *Mist1* target genes (*Rab3D* and *Rab26*) are also up-regulated, likely contributing to cell maturation [63]. We also used parotid secretory protein (*Psp*, *Bpifa2*) as a marker of terminal differentiation since it is almost exclusively expressed in parotid acinar cells and is one of the most abundant salivary proteins. PSP is a lipid and LPS-binding salivary protein with anti-inflammatory and antimicrobial activity [64, 65]. Transfection studies (Fig 8) demonstrate for the first time that either *Xbp1* or *Mist1* can directly activate expression of the *Psp* gene, acting at binding sites either in the promoter (*Xbp1*) or in introns (*Mist1*). In the *Mist1* knockout mice both pancreatic and parotid acinar cells are severely disorganized, although some secretory granules are present and *Rab3D* mRNA is expressed at 50% of normal [17]. Our observation that either *Xbp1* or *Mist1* can activate *Psp* suggests that the incomplete disruption of the acinar phenotype in *Mist1*-null parotid may be due to the ability of *Xbp1* to ‘work around’ *Mist1* to activate terminal markers of differentiation. In this view, *Xbp1* and *Mist1* act in concert, rather than sequentially, to drive differentiation.

Additional new insights from the mRNA profiles include transient up-regulation of *Pparg* along with 18 of its downstream targets (Fig 3). Expression of these genes was low in the embryo, but then increased, particularly after birth, to peak at P5. Expression then decreased sharply to return to basal level prior to P20. *Pparg* is an important driver of adipocyte differentiation, but its role in the salivary gland has not been investigated. According to our data, both *Rxra* and *Rara* are expressed throughout acinar cell development. Either of these genes could be available for dimerization with *Pparg*. The cofactor *Pgc1α* (also termed *Ppargc1*) mRNA is strongly up-regulated following the *Pparg*-related peak, potentially through *Pparg* and RXR binding sites within the *Pgc1α* gene. *Pgc1α/β* directs maturation of cardiac myocytes [66], and could contribute to maintaining ATF6α expression during differentiation of the parotid. However, the role of the transient expression of *Pparg* and related factors in parotid differentiation remains unknown.

We have used LCM to isolate developing parotid acinar cells in a nearly comprehensive approach to identify profiles of mRNAs and miRNAs which drive terminal differentiation. Cluster analysis shows 4 stages of development with strongly increased expression of key parotid genes, including several secreted cargo proteins, in the final stage (Fig 6B). Data from 27 microarrays plus additional miRNA arrays generated a transcription factor regulatory network which provides important context for transcriptional changes which drive parotid differentiation. We have validated 8 of the edges in the proposed network, and other edges are supported by interactions reported in other tissues. Future work is needed to expand this network to include extracellular signaling pathways and epigenetic changes which are important for differentiation. Nonetheless, the genes identified in this network provide additional targets and markers for research into bioengineering or regeneration of salivary glands. Characterization of expression of these genes during radiation-induced destruction of salivary glands may contribute to a molecular understanding of the loss of salivary function.

## Supporting Information

S1 Fig LCM Cap Images. Using LCM, parotid acini were able to be isolated in both embryonic and postnatal tissue. (A) H&E stained cryosections (5 μm) of rat parotid gland from embryonic day 20 (E20) and postnatal day 25 (P25). (B) Subsequent capture of cells on LCM cap, shows acinar cell isolation without appreciable contamination of ductal cells or connective tissue.
(PDF)

S2 Fig qPCR Validation of Microarray Measurements. Taqman qRT-PCR was run on triplicate RNA samples spanning nine time points of parotid acinar differentiation, using primers that amplify *Psp*, *Xbp1*, and *Nupr1*. Expression of *Rplp2* was used for normalization. The expression profiles (plotted in Log base 10) replicate the increase in expression seen in the microarrays.
(PDF)

S3 Fig Heatmap of Genes with Quadratic Expression Pattern. Quadratic regression was used to identify mRNAs with expression profiles that significantly match a quadratic model.430 were identified as having a significant match (p-value = 0.05) and a heatmap was generated using a dissimilarity distance matrix.
(PDF)

S4 Fig Cluster Analysis of Genes with Cubic Pattern. Genes with a significant cubic trend were clustered into the above 5 expression profiles. Only 18 genes were available for clustering and most clusters have only a few genes.
(PDF)

S5 Fig Linear Regression of miRNA Expression Data. 69 miRNAs have a significant linear trend and are divided into two clusters. 55 increase in expression, while 14 decrease. Normalized CT values for each microRNA were scaled to a mean = 0 and stdev = 1 before plotting in gray. The red line traces the average expression for the cluster.
(PDF)

S6 Fig Transcription Factor Binding Sites in the Rat *Psp* Gene. (A) the *Psp* promoter contains two CCACG boxes which is a consensus sequence for *Xbp1* binding. (B) Two E-boxes flank exon 3 in the *Psp* gene which match the Mist1-binding consensus sequence.
(PDF)

S7 Fig MiR-214 Target Sites in Rat Xbp1. (A) The human miR-214 binding site in the Xbp1 3'UTR is not conserved in rat (targetscan.org). (B) However the rat sequence contains a predicted miR-214 binding site about 600 bp upstream.
(PDF)

S1 Table Cloning Primers. Cloning primers used for luciferase based assays.
(PDF)

S2 Table Enrichment Analysis Cluster 1. GO enrichment analysis of DE cluster #1 (Fig 2).
(XLSX)

S3 Table Enrichment Analysis Cluster 2. GO enrichment analysis of DE cluster #2 (Fig 2).
(XLSX)

S4 Table Transcription Factor Enrichment Cluster 2. Enrichment of transcription factor targets in DE cluster #2 (Fig 2).
(PDF)

S5 Table Enrichment Analysis Cluster 4. GO enrichment analysis of DE cluster #4 (Fig 2).
(XLSX)

S6 Table Enrichment Analysis Quadratic Cluster 1. GO enrichment analysis of quadratic cluster #1 (Fig 4).
(XLSX)

S7 Table Enrichment Analysis Quadratic Cluster 7. GO enrichment analysis of quadratic cluster #7 (Fig 4).
(XLSX)

S8 Table Predicted miRNA Targets. Predicted target genes were generated in Targetscan for differentially expressed miRNAs.
(XLSX)

S9 Table miRNA Target Enrichment. Target gene enrichment analysis. Enrichment of DE clusters in predicted miRNA target genes.
(XLSX)
