# Correction: A Systems Biology Approach Identifies a Regulatory Network in Parotid Acinar Cell Terminal Differentiation

**DOI:** 10.1371/journal.pone.0131853

**Published:** 2015-06-25

**Authors:** The PLOS ONE Staff

There is an error in the Abstract. The sentence that reads “Transfection studies show that Xbp1Mist1 promoter.” should instead read as follows: “Transfection studies show that Xbp1 activates the Mist1 promoter.”
