# Supplementary material for: A Systems Biology Approach Identifies a Regulatory Network in Parotid Acinar Cell Terminal Differentiation
Source: PLoS One. 2015 Apr 30;10(4):e0125153. doi: 10.1371/journal.pone.0125153 (PMC4416001; doi:10.1371/journal.pone.0125153)
Supplement: S1 Fig — Using LCM, parotid acini were able to be isolated in both embryonic and postnatal tissue. (A) H&E stained cryosections (5 μm) of rat parotid gland from embryonic day 20 (E20) and postnatal day 25 (P25). (B) Subsequent capture of cells on LCM cap, shows acinar cell isolation without appreciable contamination of ductal cells or connective tissue. (PDF) [file pone.0125153.s001.pdf]

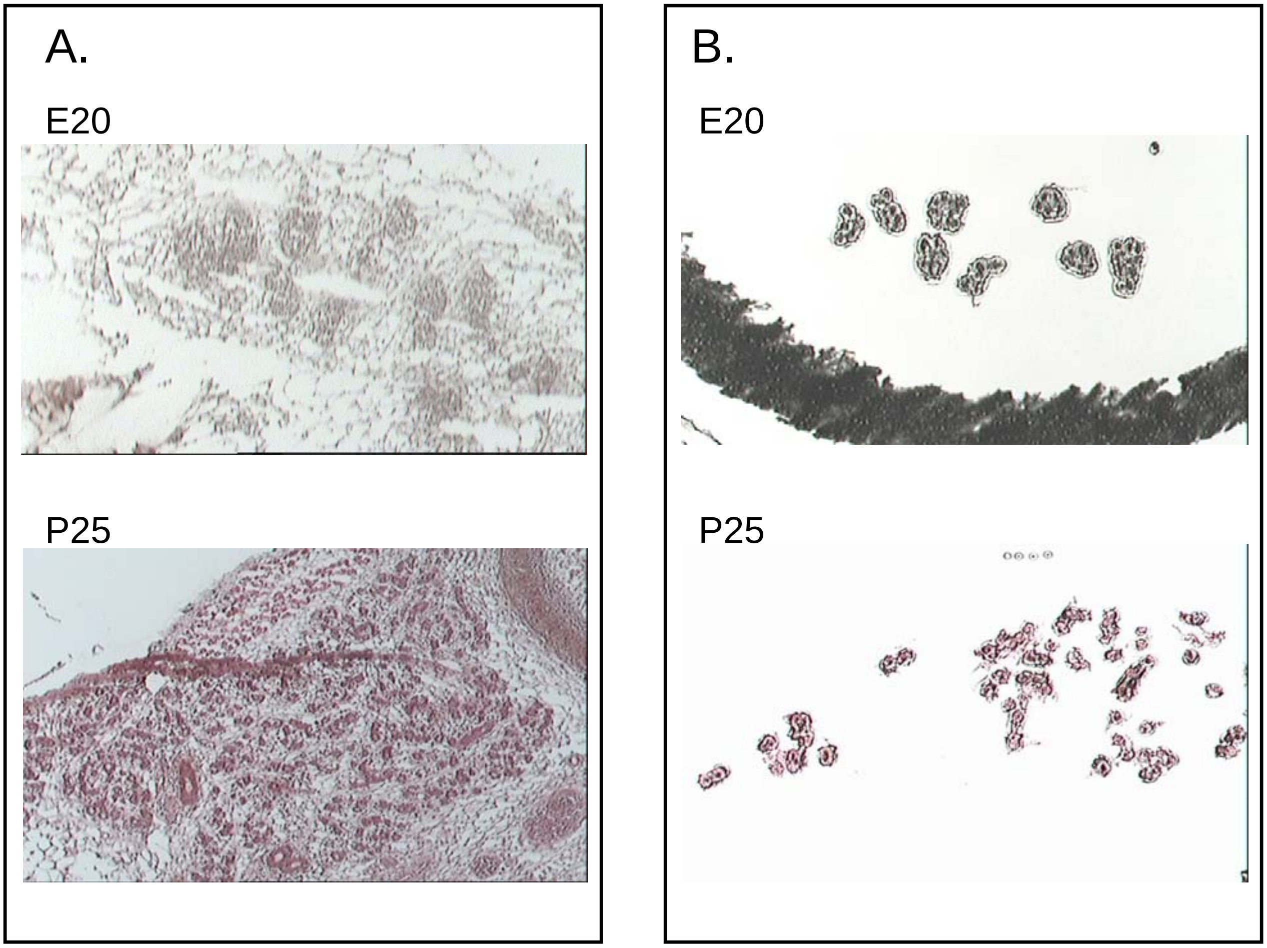

Figure S1. Using LCM, parotid acini were able to be isolated in both embryonic and postnatal tissue. (A) H&E stained cryo-sections (5µm) of rat parotid gland from embryonic day 20 (E20) and postnatal day 25 (P25). (B) Subsequent capture of cells on LCM cap, shows acinar cell isolation without appreciable contamination of ductal cells or connective tissue.
