# Supplementary material for: A Systems Biology Approach Identifies a Regulatory Network in Parotid Acinar Cell Terminal Differentiation
Source: PLoS One. 2015 Apr 30;10(4):e0125153. doi: 10.1371/journal.pone.0125153 (PMC4416001; doi:10.1371/journal.pone.0125153)

Figure S2. Microarray Validation. Taqman qRT-PCR was run on triplicate RNA samples spanning nine time points of parotid acinar differentiation, using primers that amplify *Psp*, *Xbp1*, and *Nupr1*. Expression of *Rplp2* was used for normalization. The expression profiles (plotted in Log base 10) replicate the increase in expression seen in the microarrays.

A. Expression of *Psp* during parotid gland development

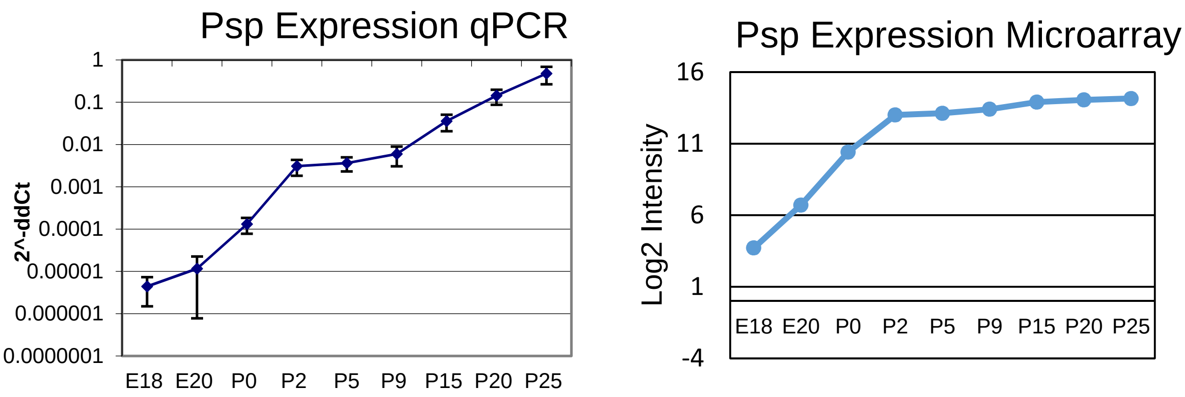

B. *Xbp1* expression during parotid gland development

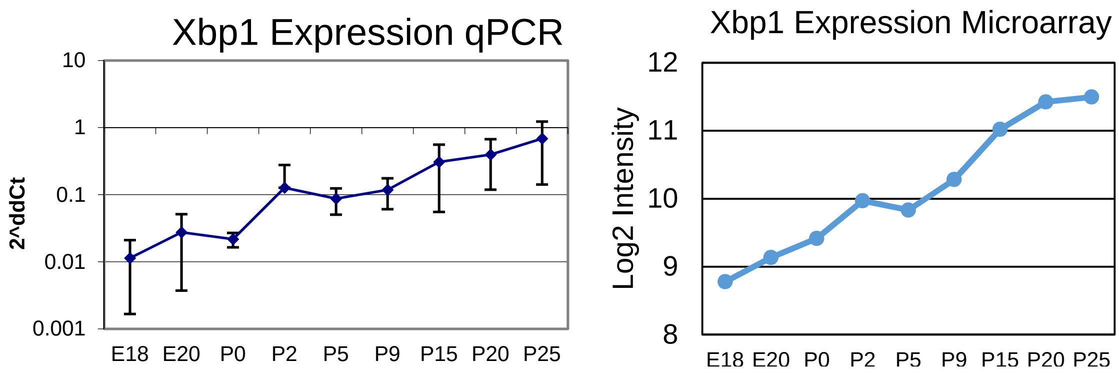

C. Expression of *Nupr1* during parotid gland development

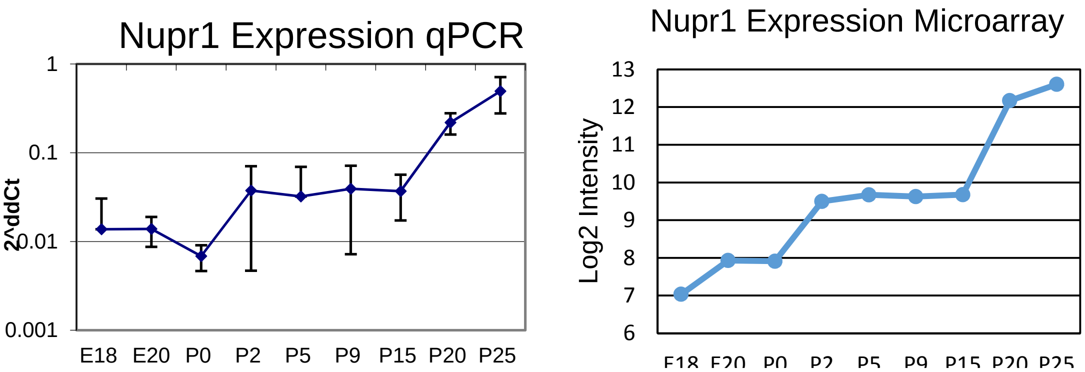

Supplement: S2 Fig — Taqman qRT-PCR was run on triplicate RNA samples spanning nine time points of parotid acinar differentiation, using primers that amplify Psp, Xbp1, and Nupr1. Expression of Rplp2 was used for normalization. The expression profiles (plotted in Log base 10) replicate the increase in expression seen in the microarrays. (PDF) [file pone.0125153.s002.pdf]
