# Supplementary material for: A Systems Biology Approach Identifies a Regulatory Network in Parotid Acinar Cell Terminal Differentiation
Source: PLoS One. 2015 Apr 30;10(4):e0125153. doi: 10.1371/journal.pone.0125153 (PMC4416001; doi:10.1371/journal.pone.0125153)

Figure S3. Quadratic regression was used to identify mRNAs with expression profiles that significantly match a quadratic model.430 were identified as having a significant match (p-value= 0.05) and a heatmap was generated using a dissimilarity distance matrix.

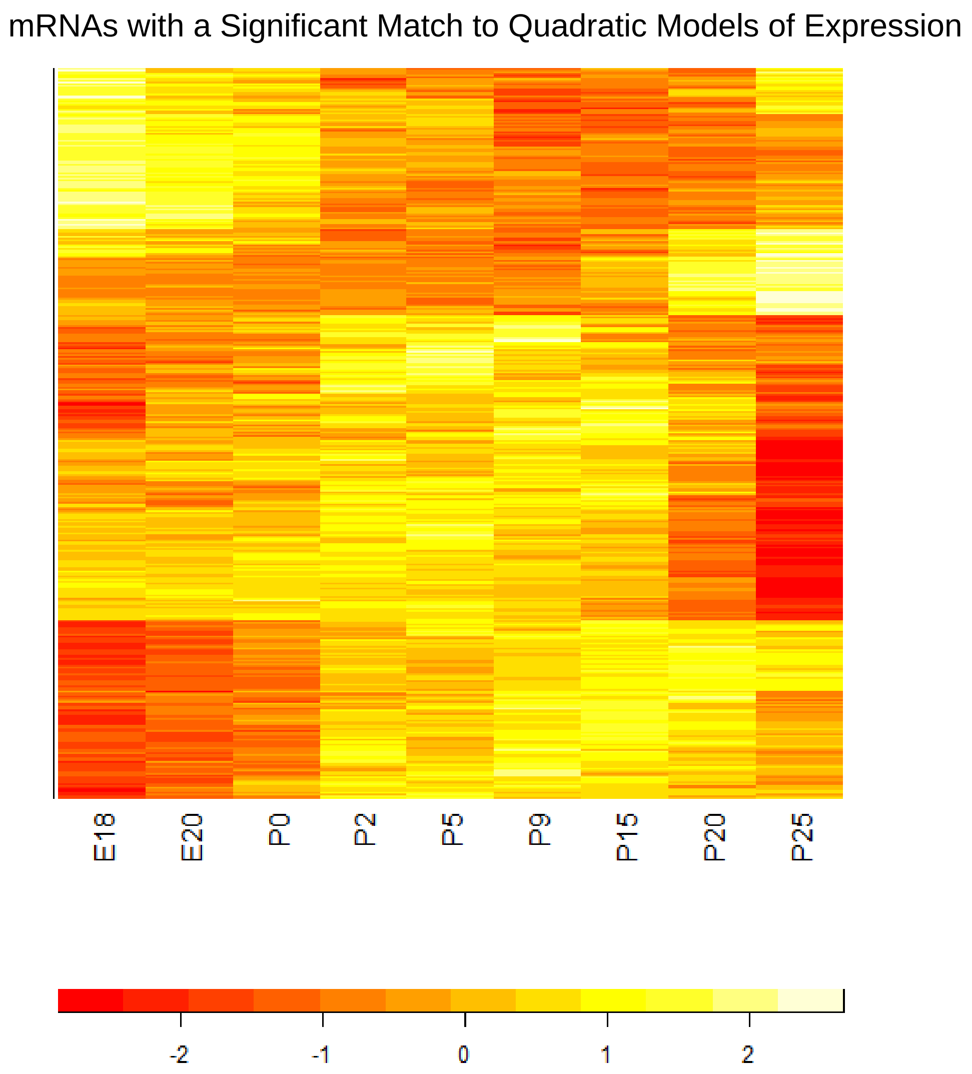

Supplement: S3 Fig — Quadratic regression was used to identify mRNAs with expression profiles that significantly match a quadratic model.430 were identified as having a significant match (p-value = 0.05) and a heatmap was generated using a dissimilarity distance matrix. (PDF) [file pone.0125153.s003.pdf]
