# Supplementary material for: A Systems Biology Approach Identifies a Regulatory Network in Parotid Acinar Cell Terminal Differentiation
Source: PLoS One. 2015 Apr 30;10(4):e0125153. doi: 10.1371/journal.pone.0125153 (PMC4416001; doi:10.1371/journal.pone.0125153)
Supplement: S4 Fig — Genes with a significant cubic trend were clustered into the above 5 expression profiles. Only 18 genes were available for clustering and most clusters have only a few genes. (PDF) [file pone.0125153.s004.pdf]

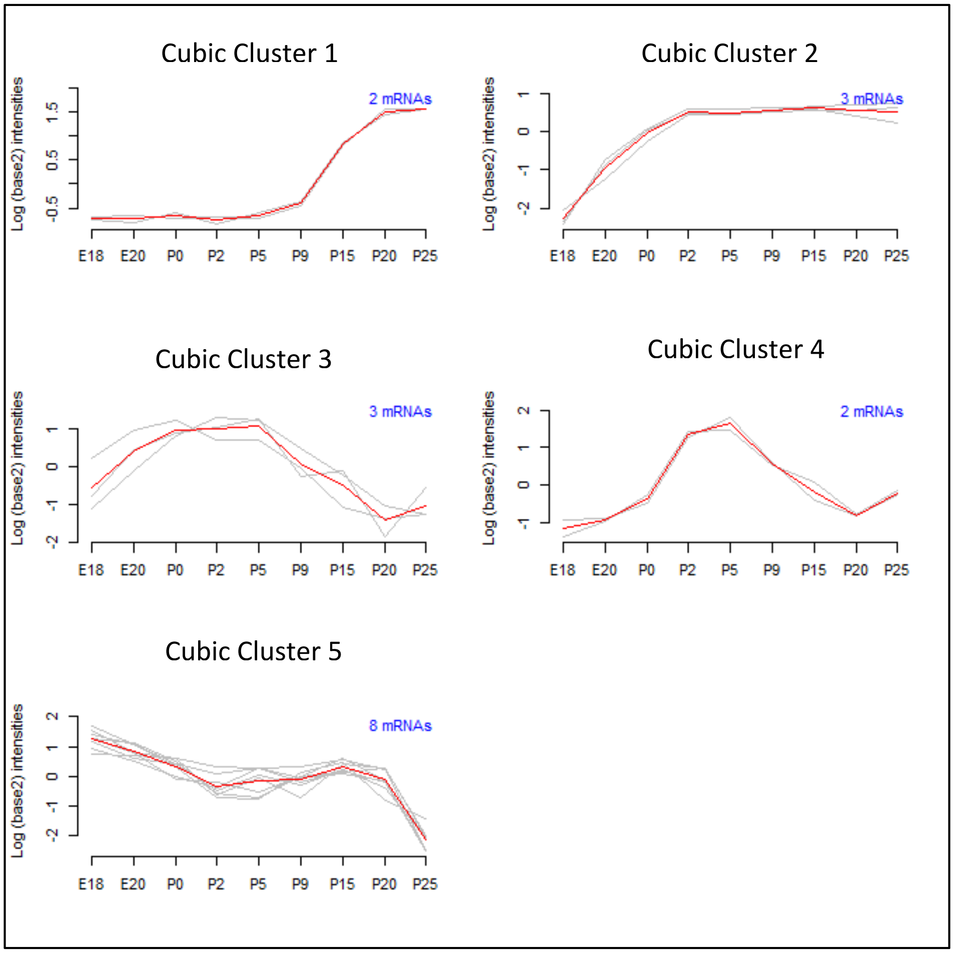

Figure S4. Clustering mRNAs with a Significant Cubic Trend. Genes with a significant cubic trend were clustered into the above 5 expression profiles. Only 18 genes were available for clustering and most clusters have only a few genes.
