# Supplementary material for: A Systems Biology Approach Identifies a Regulatory Network in Parotid Acinar Cell Terminal Differentiation
Source: PLoS One. 2015 Apr 30;10(4):e0125153. doi: 10.1371/journal.pone.0125153 (PMC4416001; doi:10.1371/journal.pone.0125153)
Supplement: S5 Fig — 69 miRNAs have a significant linear trend and are divided into two clusters. 55 increase in expression, while 14 decrease. Normalized CT values for each microRNA were scaled to a mean = 0 and stdev = 1 before plotting in gray. The red line traces the average expression for the cluster. (PDF) [file pone.0125153.s005.pdf]

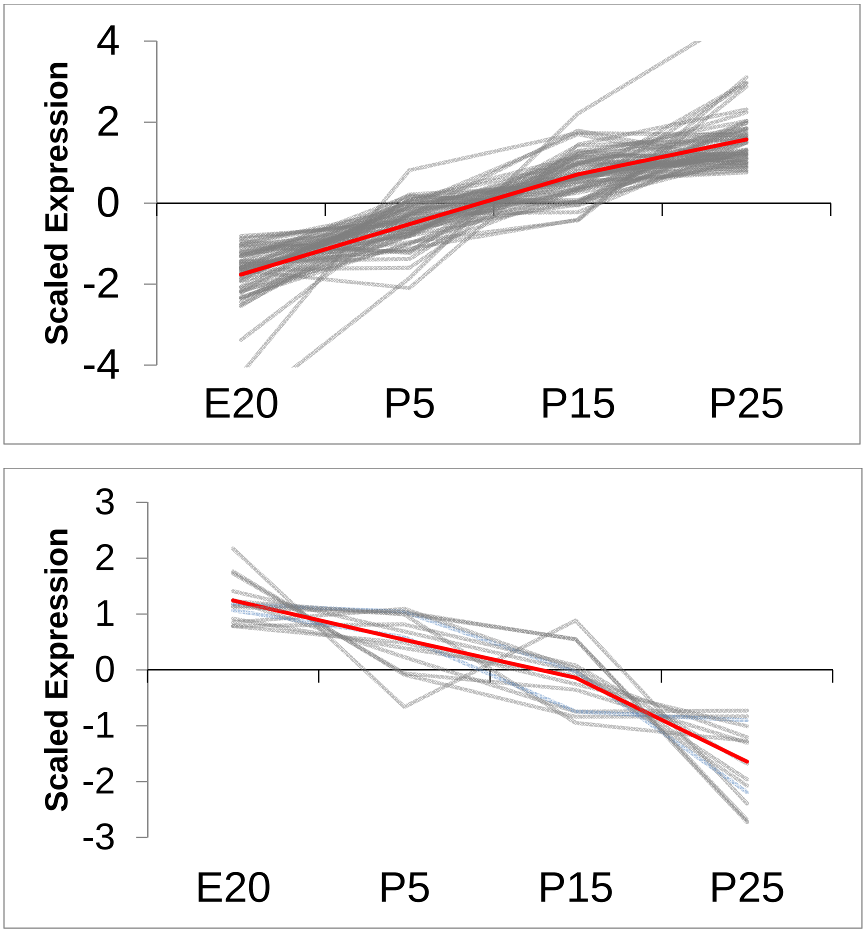

Figure S5. miRNA's with a significant linear trend. 69 miRNAs have a significant linear trend and are divided into two clusters. 55 increase in expression, while 14 decrease. Normalized CT values for each microRNA were scaled to a mean=0 and stdev=1 before plotting in gray. The red line traces the average expression for the cluster.
