# Supplementary material for: A Systems Biology Approach Identifies a Regulatory Network in Parotid Acinar Cell Terminal Differentiation
Source: PLoS One. 2015 Apr 30;10(4):e0125153. doi: 10.1371/journal.pone.0125153 (PMC4416001; doi:10.1371/journal.pone.0125153)
Supplement: S6 Fig — (A) the Psp promoter contains two CCACG boxes which is a consensus sequence for Xbp1 binding. (B) Two E-boxes flank exon 3 in the Psp gene which match the Mist1-binding consensus sequence. (PDF) [file pone.0125153.s006.pdf]

A.

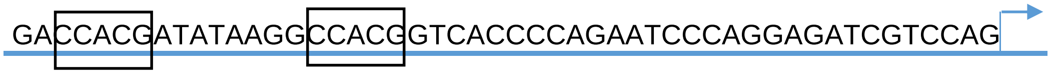

B.

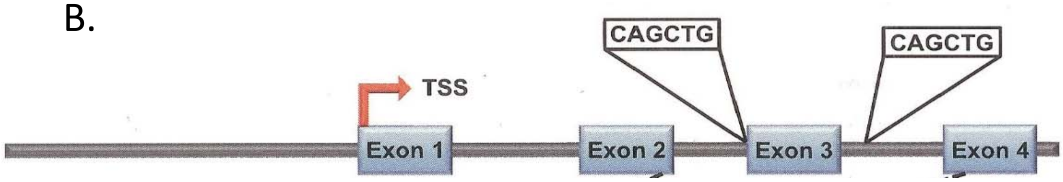

Figure S6. Predicted transcription factor binding sites. A: the *Psp* promoter contains two CCACG boxes which is a consensus sequence for *Xbp1* binding. B. Two E-boxes flank exon 3 in the *Psp* gene which match the Mist1-binding consensus sequence.
