# Supplementary material for: A Systems Biology Approach Identifies a Regulatory Network in Parotid Acinar Cell Terminal Differentiation
Source: PLoS One. 2015 Apr 30;10(4):e0125153. doi: 10.1371/journal.pone.0125153 (PMC4416001; doi:10.1371/journal.pone.0125153)
Supplement: S7 Fig — (A) The human miR-214 binding site in the Xbp1 3'UTR is not conserved in rat (targetscan.org). (B) However the rat sequence contains a predicted miR-214 binding site about 600 bp upstream. (PDF) [file pone.0125153.s007.pdf]

A.

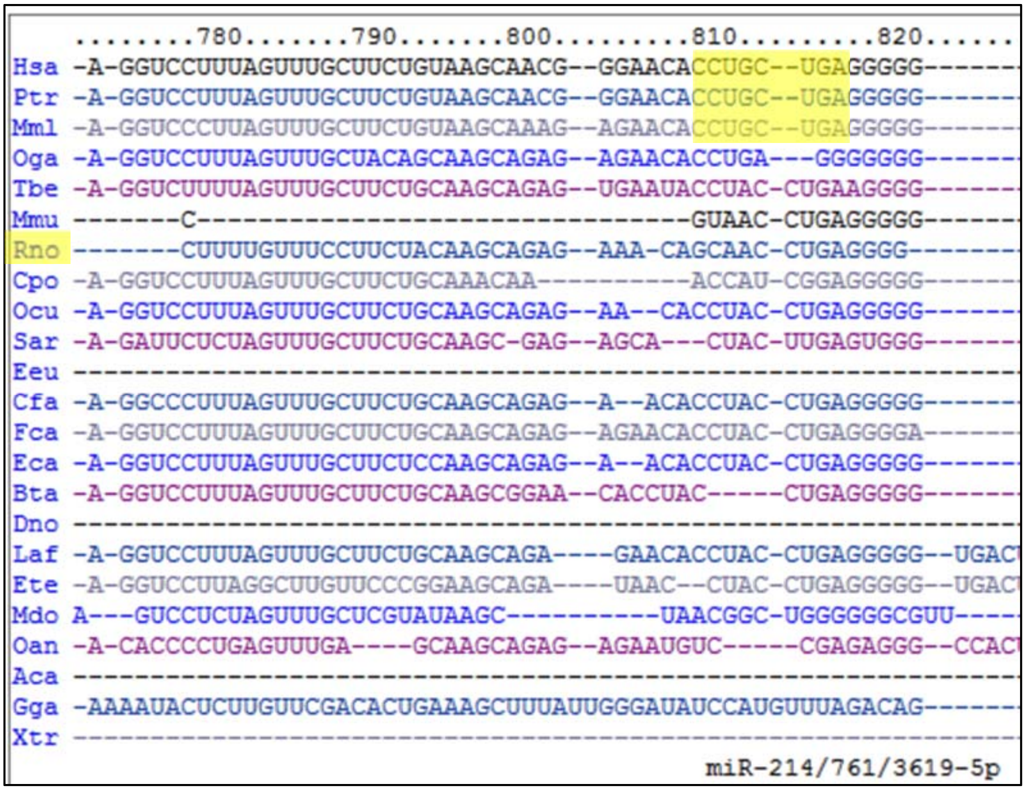

B.

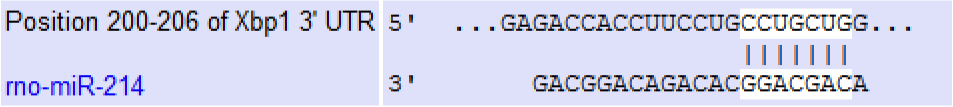

Figure S7: Rat Xbp1 3'UTR contains a miR-214 binding site. (A) The human miR-214 binding site in the Xbp1 3'UTR is not conserved in rat (targetscan.org). (B) However the rat sequence contains a predicted miR-214 binding site about 600 bp upstream.
