# Supplementary material for: A Systems Biology Approach Identifies a Regulatory Network in Parotid Acinar Cell Terminal Differentiation
Source: PLoS One. 2015 Apr 30;10(4):e0125153. doi: 10.1371/journal.pone.0125153 (PMC4416001; doi:10.1371/journal.pone.0125153)
Supplement: S1 Table — Cloning primers used for luciferase based assays. (PDF) [file pone.0125153.s008.pdf]

Target Gene	Primers
Sox11 3’UTR	For: 5' cacctctagaATAGAGTTTGCATGCCAGCG 3'<br>Rev: 5' acaaggccggccacaattcgaaCCTCTGTGAAAAACTCCTGC 3'<br>For: 5’ caccttcgaaGCATAGGCAAGGTATAGAGG 3’<br>Rev: 5’ caccggccggccGAGATCCGTCATGATACGAC 3’
Xbp1 3’UTR	For: 5’ cacctctagaTCTTAGAGATCCCCTCTGAG 3’<br>Rev: 5’ caccggccggccGCCAGGCTGAACGATAACTG 3’
Klf4 3’UTR	For: 5’ cacctctagaATTCCACATCGTGGACATGAC 3’<br>Rev: 5’ caccggccggccTGCTTAAAGGCATACTTGGG 3’
s-XBP1 cDNA	For: 5' CACCGCGGCCGCATGCTTGTGGTGGCAGCGG 3'<br>Rev: 5' CACCGGGCCCGGCTCTTTAGACACTAATCAGCTGGG 3'
Mist1 Promoter<br>-515 to +39	For: 5' GGTACCGCAGCCATGTGGTTGG 3'<br>Rev: 5' CTCGAGCACGGGGGACAAGGACACG 3'
500bp PSP promoter<br>-500 to + 21	For: 5' CACCGGTACCCATTATTGCCTCCTCCCAG 3'<br>Rev : 5' GGTGCTCGAGGACAGGAAAGCCTTGTTTC 3'
1kb PSP promoter<br>-1041 to +21	For: 5' CACCCCTTCTCTCGTCACTGAAATGTTTTC 3'<br>Rev : 5' GGTGCTCGAGGACAGGAAAGCCTTGTTTC 3'
1.5kb PSP promoter	For: 5' CACCGGTACCGCTTGGCAGACATGAGATGGAAATCG 3'<br>Rev : 5' GGTGCTCGAGGACAGGAAAGCCTTGTTTC 3'
Intron PSP	For: 5' CAACTTGTCGACCTTGTGGTCTTGTGTGGC 3'<br>Rev: 5' CATTGGTCGACAGCCCAGCTTGAAGATCC 3'

Table S1. Cloning Primers
