# Supplementary material for: A Systems Biology Approach Identifies a Regulatory Network in Parotid Acinar Cell Terminal Differentiation
Source: PLoS One. 2015 Apr 30;10(4):e0125153. doi: 10.1371/journal.pone.0125153 (PMC4416001; doi:10.1371/journal.pone.0125153)
Supplement: S4 Table — Enrichment of transcription factor targets in DE cluster #2 (Fig 2). (PDF) [file pone.0125153.s011.pdf]

Network Object Name	Actual	n	R	N	Expected	Ratio	p-value	z-score	Input IDs
MIST1	5	719	9	12845	0.5038	9.925	5.654E-05	6.522	**1387212_at**
XBP1	20	719	114	12845	6.381	3.134	4.623E-06	5.573	**1371249_at**
GCR-alpha	85	719	1004	12845	56.2	1.512	6.645E-05	4.118	

Table S4. Cluster #2 is Enriched in Targets for Mist1 and Xbp1. Transcription factor enrichment analysis was run in Metacore on the 803 genes in cluster #2. Enrichment is based on the number of downstream target genes identified in the curated Metacore database. Affy rat genome array 230 was used as the background dataset.
